# LncRNA NEAT1 suppresses cellular senescence in hepatocellular carcinoma via KIF11‐dependent repression of CDKN2A

**DOI:** 10.1002/ctm2.1418

**Published:** 2023-09-26

**Authors:** Danlei Chen, Jinghao Wang, Yang Li, Chenglin Xu, Meng Fanzheng, Pengfei Zhang, Lianxin Liu

**Affiliations:** ^1^ Department of Hepatobiliary Surgery The First Affiliated Hospital of USTC Division of Life Sciences and Medicine University of Science and Technology of China Hefei Anhui China; ^2^ Anhui Province Key Laboratory of Hepatopancreatobiliary Surgery Hefei Anhui China; ^3^ Anhui Provincial Clinical Research Center for Hepatobiliary Diseases Hefei Anhui China; ^4^ Zhejiang Cancer Hospital Hangzhou Institute of Medicine Chinese Academy of Sciences Hangzhou Zhejiang China

**Keywords:** CDKN2A, cellular senescence, hepatocellular carcinoma, KIF11, NEAT1

## Abstract

**Background:**

Hepatocellular carcinoma (HCC) is the third leading cause of cancer‐related deaths worldwide. Therapeutic options for advanced HCC are limited, which is due to a lack of full understanding of pathogenesis. Cellular senescence is a state of cell cycle arrest, which plays important roles in the pathogenesis of HCC. Mechanisms underlying hepatocellular senescence are not fully understood. LncRNA NEAT1 acts as an oncogene and contributes to the development of HCC. Whether NEAT1 modulates hepatocellular senescence in HCC is unknown.

**Methods:**

The role of NEAT1 and KIF11 in cellular senescence and tumor growth in HCC was assessed both in vitro and in vivo. RNA pulldown, mass spectrometry, Chromatin immunoprecipitation (ChIP), luciferase reporter assays, RNA FISH and immunofluorescence (IF) staining were used to explore the detailed molecular mechanism of NEAT1 and KIF11 in cellular senescence of HCC.

**Results:**

We found that NEAT1 was upregulated in tumor tissues and hepatoma cells, which negatively correlated with a senescence biomarker CDKN2A encoding p16INK4a and p14ARF proteins. NEAT1 was reduced in senescent hepatoma cells induced by doxorubicin (DOXO) or serum starvation. Furthermore, NEAT1 deficiency caused senescence in cultured hepatoma cells, and protected against the progression of HCC in a mouse model. During senescence, NEAT1 translocated into cytosol and interacted with a motor protein KIF11, resulting in KIF11 protein degradation and subsequent increased expression of CDKN2A in cultured hepatoma cells. Furthermore, KIF11 knockdown caused senescence in cultured hepatoma cells. Genetic deletion of Kif11 in hepatocytes inhibited the development of HCC in a mouse model.

**Conclusions:**

Conclusively, NEAT1 overexpression reduces senescence and promotes tumor progression in HCC tissues and hepatoma cells, whereas NEAT1 deficiency causes senescence and inhibits tumor progression in HCC. This is associated with KIF11‐dependent repression of CDKN2A. These findings lay the foundation to develop potential therapies for HCC by inhibiting NEAT1 and KIF11 or inducing senescence.

## Graphical abstract

1. NEAT1 is released from the paraspeckle and exported to the cytoplasm during ROS stress‐induced cellular senescence in HCC.

2. ROS stresses induce the nuclear export of NEAT1 to interact with KIF11 and lead to KIF11 degradation to promote cellular senescence in HCC.

3. KIF11 down‐regulation drives cellular senescence of HCC via transcriptional activation and DNA

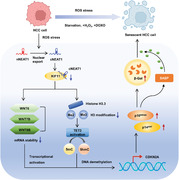

## 1 INTRODUCTION

Hepatocellular carcinoma (HCC) is the most common primary liver cancer, which is the third leading cause of cancer‐related deaths in the world.^1^ HCC is highly malignant, metastatic and recurrent. The chemotherapy options and targeted therapies for advanced HCC are still limited.^2^, ^3^ This is due to lacking full understanding of pathological mechanisms that contribute to the malignant progression of HCC.

Cellular senescence is a state of cell cycle arrest with enlarged morphology and senescence‐associated secretory phenotype (SASP).^4^ Senescence is regulated by the p16^INK4A^/pRb and p14^ARF^/p53 pathways.^5^ Cyclin‐dependent kinase inhibitor 2A (CDKN2A) gene codes for two proteins (p16^INK4a^ and p14^ARF^).^6^ p16^INK4a^ inhibits CDK4 and CDK6,^7^ while p14^ARF^  is the inhibitor of MDM2 and activates the p53 tumour suppressor.^8^ Cellular senescence is generally considered as a potential mechanism to inhibit tumour development or increase their drug sensitivity,^9^ and targeting 'cellular senescence' will also become a new anti‐cancer approach.^10^

Hepatocellular senescence has a potentially protective role against the occurrence or development of HCC.^11^ Inducing hepatocellular senescence led to growth arrest and is crucial in the early control of malignant progression of HCC.^12^ A recent study suggests the 'one‐two punch' cancer therapy, which consists of therapeutics to induce tumour cell senescence and then selectively eliminate these senescent cells.^13^ This has been demonstrated in suppressing tumour growth in a variety of mouse models of liver cancer.^12^ Therefore, understanding the mechanism underlying hepatocellular senescence would help to develop novel approaches targeting senescence for the treatment of HCC.

An increasing number of senescence‐associated lncRNAs have been identified and well studied.^14^ For instance, LncRNA SENEBLOC drives p53‐mediated and p53‐unmediated pathways to suppress senescence as described in our report.^15^ Nuclear Enriched Abundant Transcript 1 (NEAT1) is a novel long non‐coding RNA (lncRNA), localises to specific nuclear structure called paraspeckles, which regulates gene expression through interaction with proteins or nucleic acids.^16^ NEAT1 RNA interacts with paraspeckle protein and is essential for paraspeckle formation and maintenance.^17^ NEAT1 is a pan‐cancer LncRNA^18^ and contributes to the development of HCC.^19^ NEAT1 promotes proliferation and metastasis of liver cancer^20^ and self‐renewal of liver cancer stem cells.^21^ Exosome LncRNA NEAT1 derived from macrophage migration inhibitory factor‐treated mesenchymal stem cells protects against doxorubicin (DOXO)‐induced cardiac senescence.^22^ Whether NEAT1 modulates hepatocellular senescence in HCC and the involved molecular mechanism have not been elucidated. We hypothesised that NEAT1 represses hepatocellular senescence during the development of HCC. Here, we found that high expression of NEAT1 inhibited hepatocellular senescence in HCC. Moreover, knockdown of NEAT1 inhibited the development of HCC in a mouse model. Mechanistically, NEAT1 suppressed cellular senescence in HCC via kinesin family member 11 (KIF11)‐dependent repression of CDKN2A.

## 2 RESULTS

### 2.1 NEAT1 is decreased during replicative and stress‐induced senescence

**FIGURE 1:**
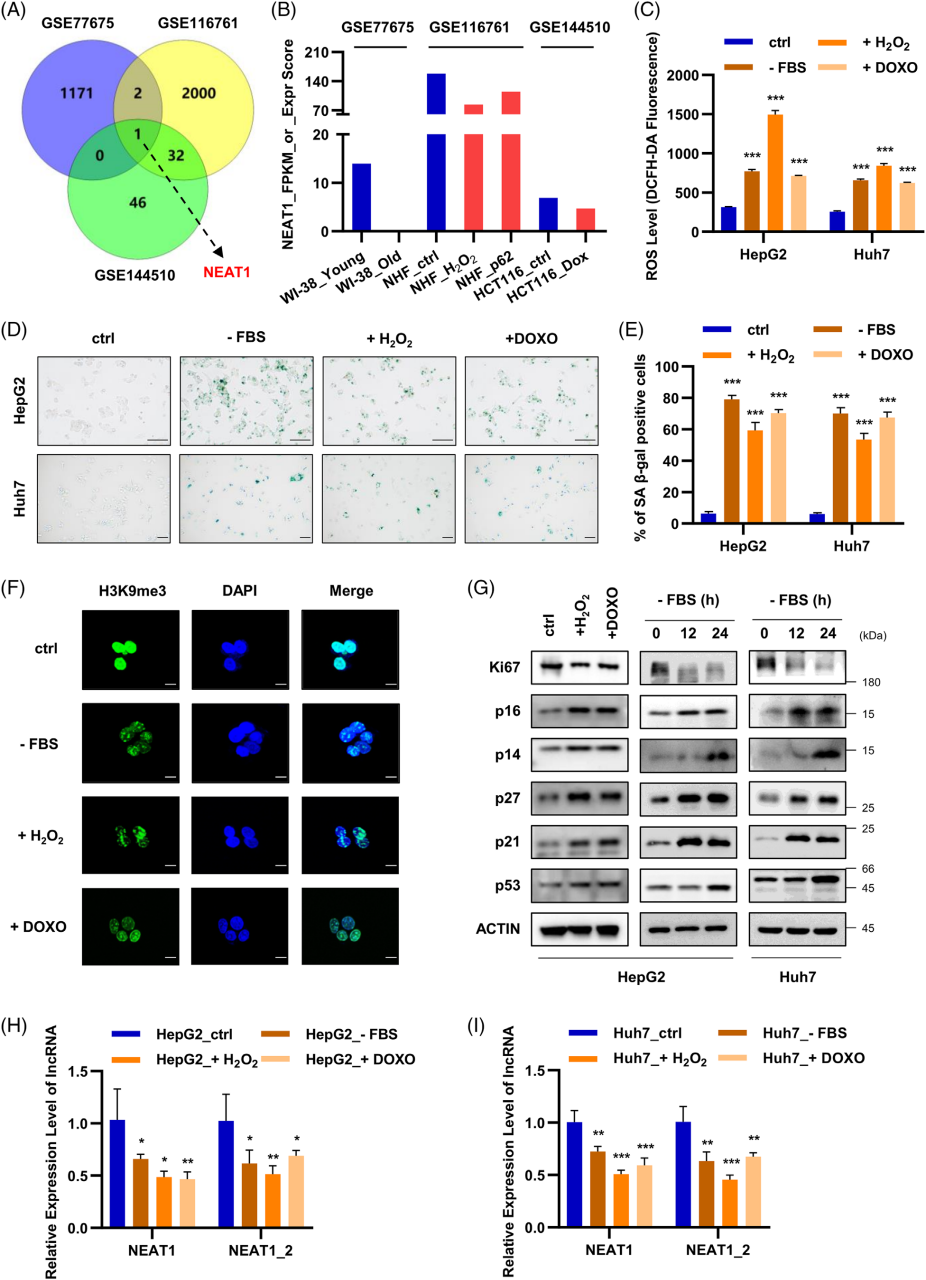
NEAT1 is down‐regulated in ROS stress‐induced senescent hepatoma cells. (A) Venn diagram of overlapping down‐regulated lncRNAs in multiple types of senescent cells in GSE77675, GSE116761 and GSE144510 datasets. GSE77675: Down‐regulated mRNAs and lncRNAs in senescent WI‐38 cells (log2FC < −5). GSE116761: Down‐regulated lncRNAs in not only replicative senescent NHFs but also H_2_O_2_‐treated NHFs (log2FC < −0.3). GSE144510: Down‐regulated lncRNAs in HCT116 cells treated with DOXO (1 μM), log2FC < −0.3. (B) The FPKM or expression score of NEAT1 in samples of GSE77675, GSE116761 and GSE144510 datasets. (C) ROS production level was quantified using 2,7‐dichlorofluorescin diacetate (DCFH‐DA), which measures hydroxyl and peroxyl radicals. (D) SA‐β‐gal staining of control and stress‐induced hepatoma cells. The scale bar indicates 100 μm. (E) The graph shows percentage of SA‐β‐gal positive cells. (F) H3K9me3 staining of control and stress‐induced hepatoma cells. The scale bar indicates 10 μm. (G) Western blotting were carried out to detect the changes in the expression of cellular senescence‐related markers in control and stress‐induced HepG2 cells. (H and I) qPCR showing total NEAT1 and NEAT1_2 expression levels in the control, serum‐starved, H_2_O_2_‐treated or DOXO‐treated HepG2 and Huh7 cells. Data shown are the mean ± SD (*n* ≥ 3; *p < .05, **p < .01, ***p < .001, two‐tailed *t*‐test).

We have been focusing on the role of lncRNAs in the development of cellular senescence. First, we re‐analysed and integrated the GSE77675, GSE116761 and GSE144510 datasets and tried to identify possible tumour‐related lncRNAs involved in common replicative and stress‐induced senescence. Young and old human fibroblasts are used to study replicative senescence in the GSE77675 and GSE116761 datasets. H_2_O_2_ (600 μM) and DOXO (1 μM) are used to induce premature senescence in human fibroblasts and a human colorectal carcinoma cell line (HCT116 cells) in the GSE116761 and GSE144510 datasets, respectively. Venn diagram shows the overlapping down‐regulated lncRNAs in multiple types of senescent cells in GSE77675, GSE116761 and GSE144510 datasets. Through integrated analysis, there was only one lncRNA NEAT1 that was significantly down‐regulated during the development of replicative senescence and stress‐induced cellular senescence (Figures 1A and B).

To further determine whether NEAT1 is reduced during stress‐induced senescence in hepatoma cells, both HepG2 and Huh7 cells were cultured in a serum‐free medium (starvation) for 48 h or H_2_O_2_ (100 μM) for 24 h or DOXO (100 nM) for 24 h to induce reactive oxygen species (ROS) stress. Fluorescence probe DCFH‐DA was used to detect ROS levels. As expected, serum starvation, H_2_O_2_ and DOXO treatment significantly increased ROS production in both HepG2 and Huh7 cells (Figure 1C). This is agreement with the findings that serum starvation,^23^ H_2_O_2_ ^24^, ^25^ and DOXO^26^ generate high levels of ROS.^27^ Meanwhile, these treatments also increased the proportion of senescence‐associated β‐galactosidase (SA‐β‐Gal) positive cells (Figures 1D and E) and the formation of heterochromatin foci in the nucleus of HepG2 cells (Figure 1F). Levels of other senescence biomarkers, including p16, p14, p27, p21 and p53, were also increased in hepatoma cells after these treatments (Figure 1G), suggesting p16/pRb and p14/p53 pathways are activated during ROS stress‐induced cellular senescence. This agrees with previous studies showing that ROS and starvation causes cellular senescence.^28^, ^29^, ^30^ As shown in Figure S1A, human NEAT1 is identified to contain a shorter isoform NEAT1_1 (3.76 knt) and a longer isoform NEAT1_2 (22.74 knt). Finally, we measured their expression in these liver cancer cells after these treatments. Starvation, H_2_O_2_ and DOXO treatments significantly down‐regulated the expression of NEAT1 in both HepG2 (Figure 1H) and Huh7 (Figure 1I) cells. Longer transcript NEAT1_2 of NEAT1 was also inhibited in HepG2 (Figure 1H) and Huh7 (Figure 1I) cells after these treatments. Although direct analysis of senescent and proliferative HCC cells may identify a different lncRNA for HCC senescence, our study identified NEAT1 as a representative senescent lncRNA in the common cellular senescence pathway during the malignant tumour progression. Altogether, these results demonstrate that NEAT1 is decreased in replicative senescence in fibroblasts and ROS stress‐induced senescence in cultured hepatoma cells.

### 2.2 NEAT1 is increased in clinical HCC tissues and hepatoma cells, and this negatively correlates with senescence

**FIGURE 2:**
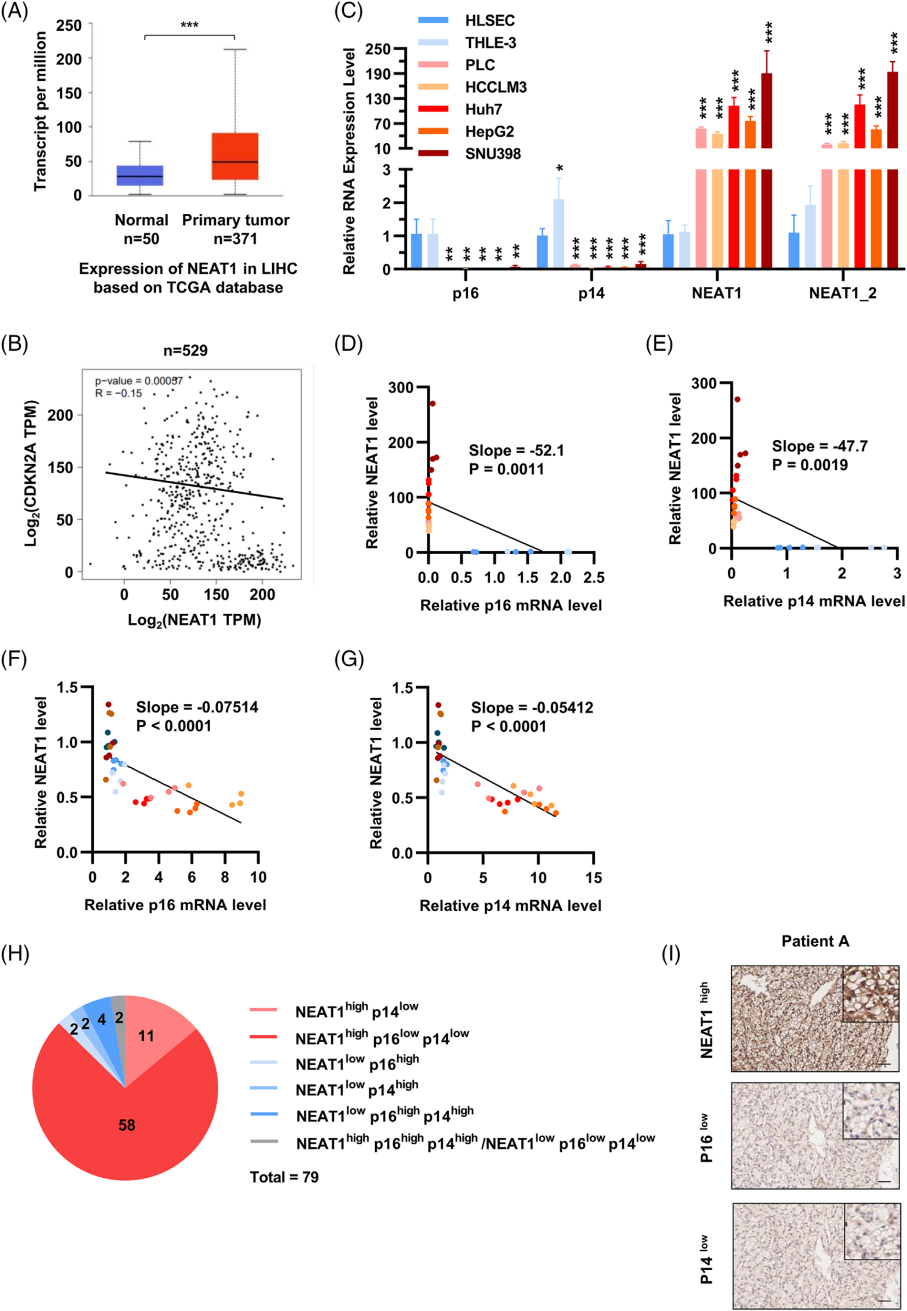
Expression level of NEAT1 and CDKN2A (p16 and p14) in HCC tissues and hepatoma cells. (A) Based on the mRNA expression profile data and clinical information in the TCGA database, NEAT1 expression in HCC tissues and associated adjacent tissues were displayed in boxplots. (B) Correlation analysis of NEAT1 and CDKN2A in clinical HCC tissues based on TCGA database. (C) Expression level of NEAT1, p16 and p14 in cultured normal liver cells (HLSEC and THLE‐3) and hepatoma cells (HCCLM3, Huh7, HepG2, SNU398). (D) Correlation analysis of NEAT1 and p16 in cultured normal liver cells (HLSEC and THLE‐3) and hepatoma cells (HCCLM3, Huh7, HepG2, SNU398). (E) Correlation analysis of NEAT1 and p14 in cultured normal liver cells (HLSEC and THLE‐3) and hepatoma cells (HCCLM3, Huh7, HepG2, SNU398). (F) Correlation analysis of NEAT1 and p16 in ROS stress‐induced normal liver cells (THLE‐3) and hepatoma cells (Huh7 and HepG2). (G) Correlation analysis of NEAT1 and p14 in ROS stress‐induced normal liver cells (THLE‐3) and hepatoma cells (Huh7 and HepG2). (H) Pie chart of different expression levels of NEAT1, p16 and p14 in clinical HCC tissues. A total of 79 clinical cases were analysed. 11 cases were NEAT1^high^ p14^low^, 58 cases were NEAT1^high^ p16^low^ p14^low^, 2 cases were NEAT1^low^ p16^high^, 2 cases were NEAT1^low^ p14^high^, 4 cases were NEAT1^low^ p16^high^ p14^high^ and 2 cases were NEAT1^high^ p16^high^ p14^high^ /NEAT1^low^ p16^low^ p14^low^. (I) IHC pictures NEAT1^high^ p16^low^ p14^low^ expression in patient A. The scale bar indicates 50 μm. Data shown are the mean ± SD (*n* ≥ 3; *p < .05, **p < .01, ***p < .001, two‐tailed *t*‐test).

To study the levels of NEAT1 in clinical HCC tissues and its association with hepatocellular senescence, we first employed the TCGA database and GTEx datasets and analysed NEAT1 and CDKN2A expression in clinical HCC tissues. As shown in Figure 2A, NEAT1 was highly expressed in clinical HCC tissues compared to their adjacent tissues. Furthermore, NEAT1 expression negatively correlated with CDKN2A levels in clinical HCC tissues (Figure 2B). We then detected the expression of NEAT1, p16 and p14 in cultured normal liver cells (i.e., HLSEC and THLE‐3) and different hepatoma cell lines (i.e., HCCLM3, Huh7, HepG2 and SNU398). Consistent with the clinical findings, NEAT1 was highly expressed in cultured hepatoma cells compared with normal liver cells (Figure 2C). Additionally, NEAT1 negatively correlated with p16/p14 levels in both normal liver cells and hepatoma cells without treatments (Figures 2D, E and S1B, C).

Then, we determined the expression of NEAT1, p16 and p14 in starvation or DOXO‐treated normal liver epithelial cells (THLE‐3) and hepatoma cells (HepG2 and Huh7). After these treatments, the expression of NEAT1 was decreased, whereas p16 and p14 were increased in these cells (Figure S1D). Interestingly, these effects were apparent in liver cancer cell lines compared with normal liver cell lines (Figure S1D). Similarly, NEAT1 expression negatively correlated with p16 and p14 levels in starvation or DOXO‐treated normal liver cells and hepatoma cells (Figures 2F, G and S1E, F). We then analysed the correlation among the expression levels of NEAT1 lncRNA, p16 protein and p14 protein in clinical HCC tissues. A total of 79 cases of HCC tissues were stained by in situ hybridisation (ISH) or immunohistochemical (IHC) and scored according to staining intensity. As shown in the pie chart (Figures 2H and S1G), 11 cases were NEAT1^high^ p14^low^, 58 cases were NEAT1^high^ p16^low^ p14^low^. IHC pictures showed different expression of NEAT1, p16 and p14 in clinical HCC tissues (Figures 2I and S1H). Totally, in about 87% (69 in 79 cases) of HCC patients, the expression of NEAT1 is higher, while p16 or (and) p14 is low. These results indicate that the high expression of NEAT1 inhibits the cellular senescence in the liver of patients with HCC. Altogether, these results suggest that NEAT1 is increased in clinical HCC tissues and hepatoma cells, which negatively correlates with senescence.

### 2.3 NEAT1 knockdown leads to cellular senescence and restrains HCC progression

**FIGURE 3:**
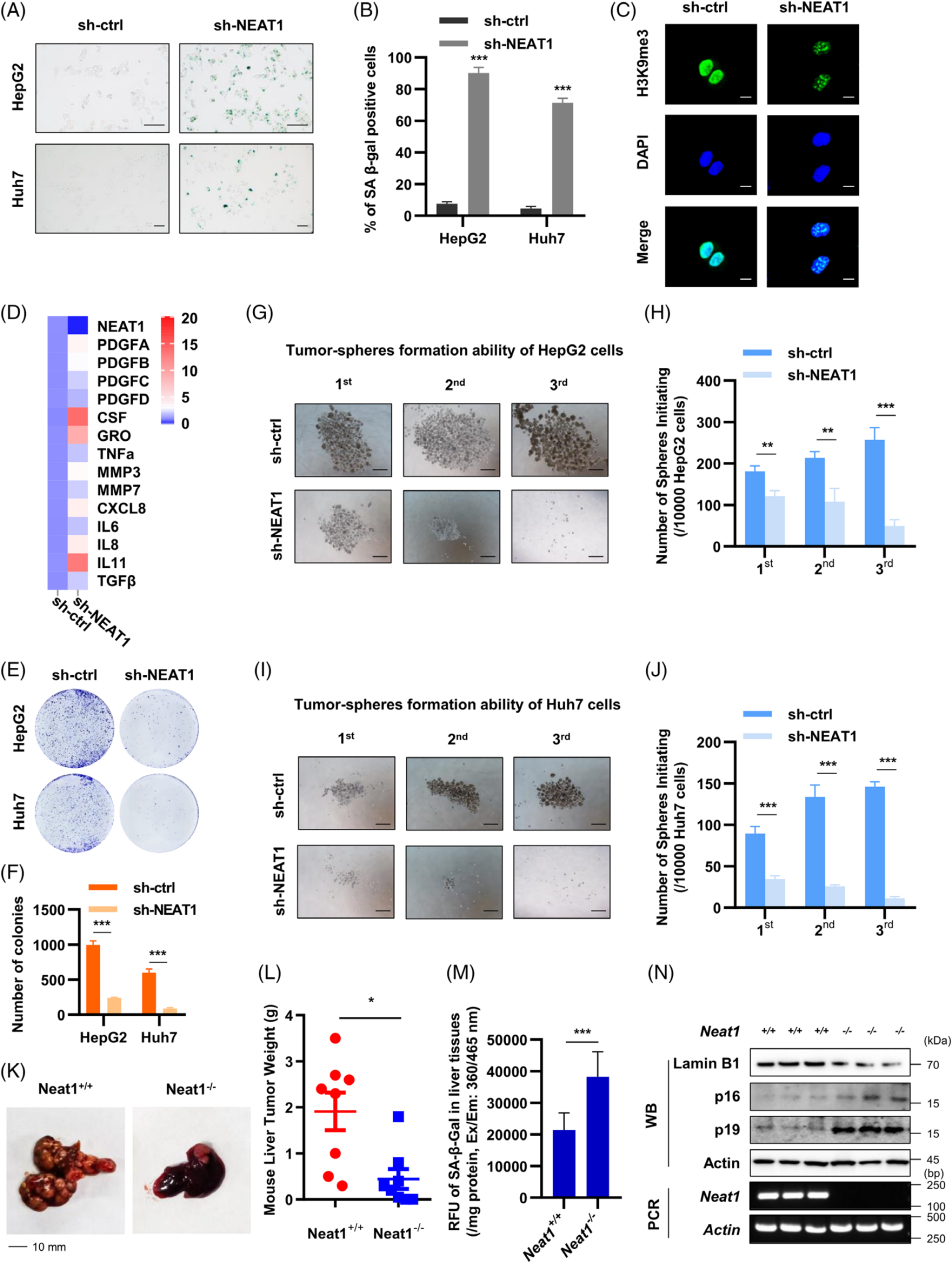
Effect of NEAT1 on cellular senescence and tumour growth of HCC. SA‐β‐galactosidase staining (A and B) and heterochromatin foci formation (C) were detected in sh‐ctrl or sh‐NEAT1 HepG2 and Huh7 cells. In A, the scale bar indicates 100 μm. In C, the scale bar indicates 10 μm. (D) SASP in sh‐ctrl or sh‐NEAT1 HepG2 cells was detected by qPCR. The average of relative expression levels were shown in the heatmap (*n* = 3). (E and F) Colony formation assay was used to detect the clone formation ability of sh‐ctrl or sh‐NEAT1 HepG2 and Huh7 cells. The data were presented in column graph. (G–J) Tumour‐spheres formation assay was used to detect the self‐renewal ability of sh‐ctrl or sh‐NEAT1 HepG2 and Huh7 cells. The scale bar indicates 500 μm. (K–M) Liver tumour (K), tumour weight (L) and liver SA‐β‐Gal activity (M) of *Neat1^+/+^* and *Neat1^−/−^* mouse, each group contains eight mice. (N) mRNA level of Neat1 and protein levels of p16, p19 and Lamin B1 in liver tumour from *Neat1^+/+^* and *Neat1^−/−^* mouse (*n* = 8). Data shown are the mean ± SD (*n* ≥ 3; *p < .05, **p < .01, ***p < .001, two‐tailed *t*‐test).

To determine the effect of NEAT1 on senescence in hepatoma cells, we used lentiviral system to stably knockdown or overexpress NEAT1 in HepG2 and Huh7 cells. We found that NEAT1 deficiency increased the proportion of SA‐β‐Gal positive hepatoma cells (Figures 3A and B) and the formation of heterochromatin foci in the nucleus of HepG2 cells (Figure 3C). In contrast, NEAT1 overexpression rescued senescent phenotype caused by serum starvation (Figures S2A–C). SASP is a dynamic phenotype consist of inflammatory cytokines, chemokines, growth factors and exosomes.^31^, ^32^ We found that knocking down NEAT1 activated SASP, evidenced by increased levels of PDGF family, cytokines (CSF, CRO, TNF‐α, TGF‐β, IL‐6, IL‐8 and IL‐11) and chemokines (CXCL8) (Figure 3D). NEAT1 overexpression reduced the levels of those SASP factors caused by serum starvation (Figure S2D). Therefore, NEAT1 represses senescence in cultured hepatoma cells.

Above data showed that various ROS stresses reduced NEAT1 expression to induce cellular senescence in HepG2 and Huh7 cell (Figures 1D–I 3A–D and S2A–D). However, HepG2 cells express functional, wild‐type p53, whereas Huh7 cells express transcriptionally inactive, Y220C‐mutated p53.^33^, ^34^ In addition, Neat1 has been reported as a p53‐inducible lincRNA.^35^ Hence, it was assumed that ROS stresses might reduce NEAT1 expression and induce senescence in a p53‐independent manner. Furtherly, HCT 116 p53^+/+^ and p53^−/−^ cells were treated with serum‐free medium or H_2_O_2_. Both cells showed significant cellular senescence, higher CDKN2A (p16) expression and lower NEAT1 expression under ROS stresses (Figures S2E–G). These data prove that ROS induce lower NEAT1 expression and cellular senescence in a p53‐independent manner in cancer cells.

We then determined the role of NEAT1 in HCC development. First, we measured tumour growth in clone formation and tumour‐spheres formation experiments using control and NEAT1 knockdown hepatoma cells. We found that knockdown NEAT1 inhibited clone formation (Figures 3E and F) and tumour‐spheres formation of hepatoma cells (Figures 3G–J). This indicates that knockdown NEAT1 reduces self‐renewal ability of cultured hepatoma cells. Next, we used Neat1 knockout (*Neat1^−/−^*) mice and their WT littermates to determine their tumour growth of HCC. Primary liver cancer was established by injecting plasmids mixture encoding pT3‐c‐MYC, pX330‐sg‐p53‐cas9 and pT2‐SB13 transposase into the tail vein of experimental and control group mice as described previously.^36^, ^37^ We found that malignant degree (Figure 3K) and weight (Figure 3L) of liver tumours in *Neat1^−/−^* group were lower than those in *Neat1^+/+^* (WT) mice. Furthermore, SA‐β‐Gal activity, p16 and p19 (p14 in human) were up‐regulated in liver cancer tissues of the *Neat1^−/−^* mice compared with WT littermates (Figures 3M and N). As shown in Figure 3N, the liver tissues of the *Neat1^−/−^* mice showed loss of Lamin B1, compared with those of WT littermates. These results indicate that NEAT1 deletion activates the p16 and p14 signalling pathways, promotes cellular senescence and inhibits development of HCC.

### 2.4 NEAT1 is translocated into cytosol and interacts with KIF11 to enhance the KIF11 protein degradation during ROS stress‐induced cellular senescence in hepatoma cells

**FIGURE 4:**
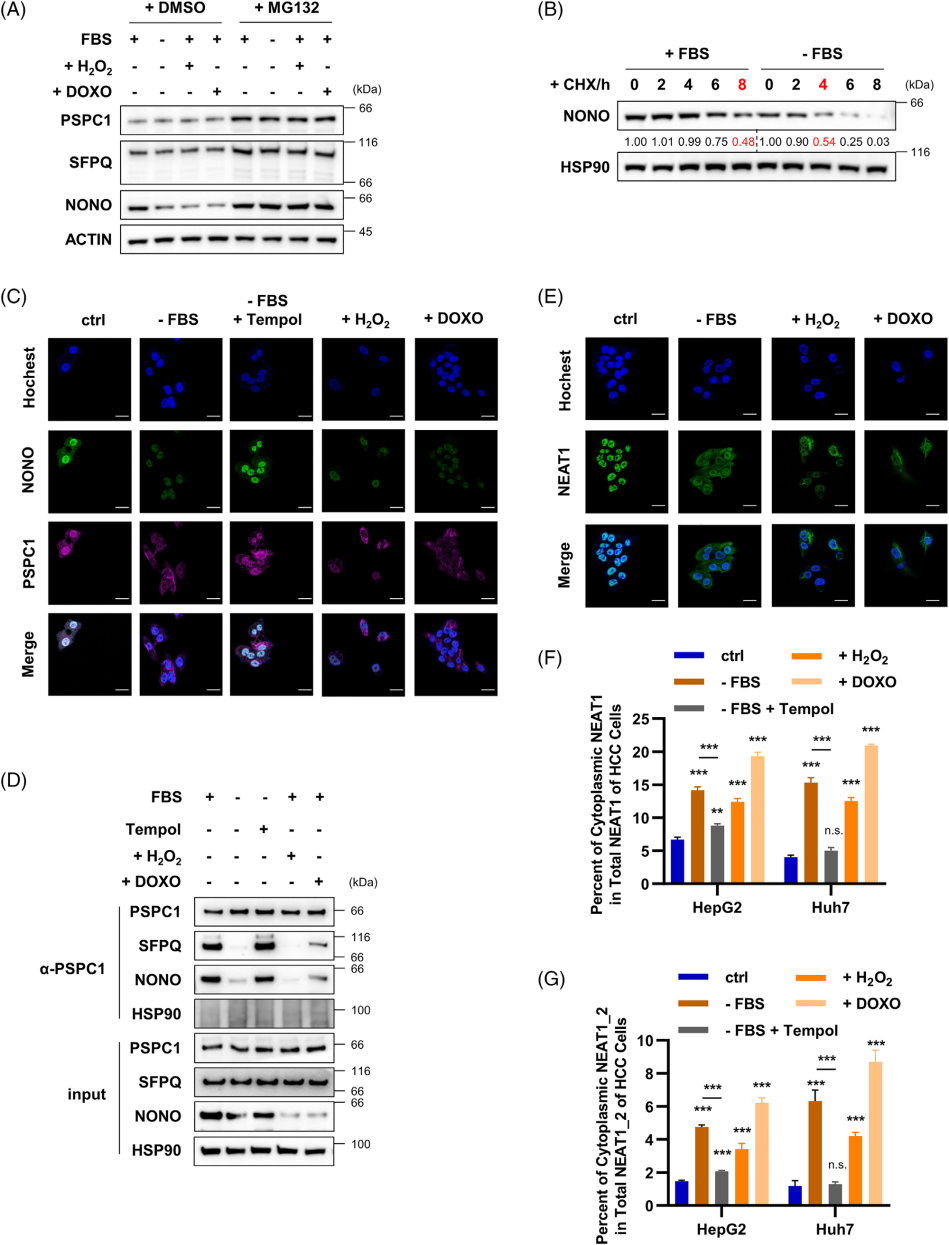
NEAT1 exits the nucleus and enters the cytoplasm in ROS stress‐induced senescent hepatoma cells. (A) Cells were treated with or without MG132 (20 μM) for 6 h. Cell lysates were analysed by Western blotting to analyse NONO, PSPC1, SFPQ and ACTIN protein levels. (B) The control and senescent cells were treated with CHX (50 μg/mL) for the indicated periods of time. Cell lysates were then analysed by Western blotting to examine the half‐life of NONO protein. Furtherly, serum‐starved cells were treated with or without Tempol (3 mM) for 12 h before analysation. (C) Co‐localisation of paraspeckle proteins, NONO (Green) and PSPC1 (Purple) were assayed by IF. The location of paraspeckles was obtained by merging two signals of NONO and PSPC1. The position of the nucleus is labelled by Hochest (Blue). The scale bar indicates 20 μm. (D) The cell lysates of control or senescent HepG2 cells subjected for co‐IP with anti‐PSPC1 antibodies to detect the protein–protein interactions of paraspeckles by WB. (E) The subcellular co‐localisation of NEAT1 (Green) was analysed by RNA‐FISH. The position of the nucleus is labelled by Hochest (Blue). The scale bar indicates 20 μm. (F and G) The cytoplasmic ratios of NEAT1 and NEAT1_2 were analysed by qPCR. Data shown are the mean ± SD (*n* ≥ 3; *p < .05, **p < .01, ***p < .001, two‐tailed *t*‐test).

LncRNA NEAT1 and proteins NONO, PSPC1 and SFPQ are the core components of intracellular subcellular paraspeckle.^16^ To investigate the down‐regulation of NEAT1 in senescent cells caused by excessive accumulation of ROS stress, we first studied the changes of the paraspeckles. We found that ROS stress, including serum starvation, H_2_O_2_ or DOXO treatments, induced cellular senescence and resulted in decreased NONO protein levels (Figure 4A). These effects were attenuated when cells were incubated with a proteasome inhibitor MG132 (Figure 4A), suggesting a proteasome‐dependent degradation. Serum starvation reduced the half‐life of NONO protein from about 8 h in normal culture to about 4 h under cycloheximide (CHX) treatment (Figure 4B). Furthermore, as shown in Figure 4C, all these ROS stress led to inhibition of paraspeckle assembly, and this was rescued by a ROS scavenger Tempol. Besides, the co‐IP with PSPC1‐specific antibody was performed in ROS stress‐induced senescent HepG2 cells, together with the Tempol‐rescued HepG2 cells. As shown in Figure 4D, the protein–protein interactions of paraspeckles were weakened in senescent cells, which was rescued by Tempol treatment. These data further demonstrate that paraspeckle assembly was inhibited in the ROS stress‐induced senescent cells. We reported that paraspeckle depolymerisation causes nucleus‐cytoplasm redistribution of NEAT1.^38^ We found that NEAT1 was translocated from the nucleus to the cytoplasm in senescent HepG2 cells (Figure 4E). Nucleus‐cytoplasm separation and RNA relative quantification of ROS stress‐induced senescent HepG2 cells showed that a 3‐to‐5‐fold increase in the proportion of NEAT1 in cytoplasm (Figures 4F and S3A, B). The longer transcript of NEAT1 and NEAT1_2 was analysed separately and showed similar changes (Figures 4G and S3A, B). Altogether, NEAT1 translocated from nucleus to cytoplasm in ROS stress‐induced senescent hepatoma cells.

**FIGURE 5:**
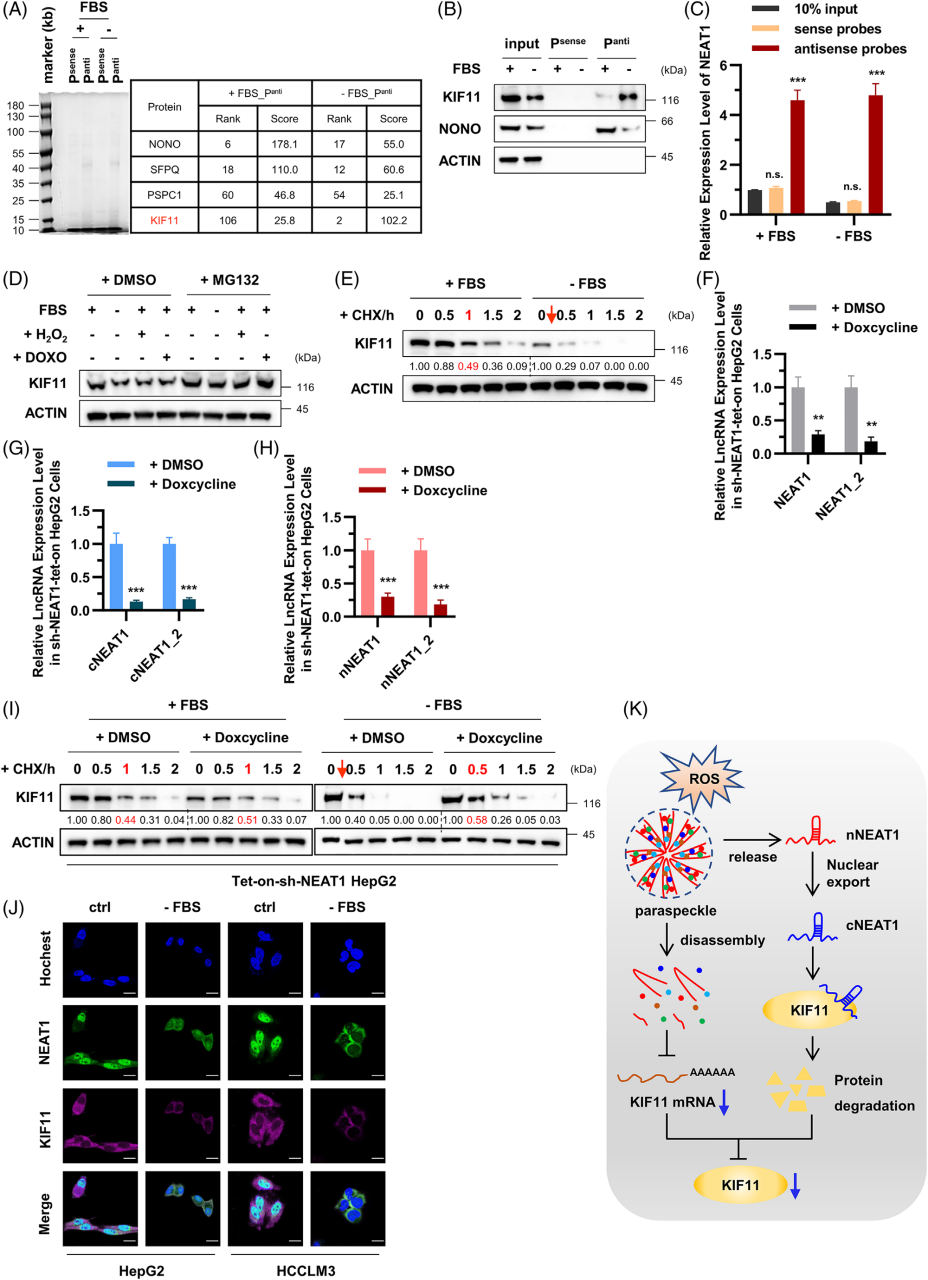
NEAT1 binds to KIF11 in cytoplasm in ROS stress‐induced senescent hepatoma cells. (A) RNA‐protein pull‐down assays were conducted against HepG2 cell lysates with biotin‐labelled sense (control) or antisense (test) NEAT1 probes. The identified proteins, interacted with NEAT1, were presented by mass spectrometry as KIF11, PSPC1, SFPQ and NONO. (B and C) HepG2 cell lysates were incubated with in vitro synthesised biotin‐labelled sense or antisense DNA probes against NEAT1 for the biotin pull‐down assay. The precipitates from the pull‐down underwent Western blotting and real‐time qPCR analyses to examine the levels of indicated proteins and lncRNA NEAT1, respectively. (D) Control, serum‐starved, H_2_O_2_‐treated and DOXO‐treated cells were following treated with or without MG132, and then were used to detect KIF11 protein levels. (E) Control, serum‐starved, H_2_O_2_‐treated and DOXO‐treated cells were treated with CHX as indicated time, and then were used to detect the KIF11 protein half‐life. (F) HepG2 cells were infected with lentiviruses expressing doxycycline‐induced NEAT1 shRNA. The knockdown efficiency was analysed by qPCR. (G and H) The cytoplasmic and nuclear ratios of NEAT1 and NEAT1_2 in indicated cells were analysed by qPCR. (I) HepG2‐tet‐on‐sh‐NEAT1 cells, cultured in completed medium or serum‐free medium, were treated with or without doxycycline (1 μg/mL) for 24 h. These cells were following treated with or without CHX, and then were used to detect KIF11 protein half‐life by Western blotting. (J) Co‐localisation of KIF11 protein (purple) and NEAT1 lncRNA (green) were assayed by IF and FISH. The co‐localisation was obtained by merging two signals of KIF11 and NEAT1. The position of the nucleus is labelled by Hochest (Blue). The scale bar indicates 20 μm. (K) Schematic illustration showing the working model for NEAT1 in regulation of KIF11 by translocation to cytoplasm under ROS stress. Data shown are mean ± SD (*n* = 3; *p < .05, **p < .01, ***p < .001, two‐tailed *t*‐test).

To determine whether NEAT1 modulates ROS stress‐induced cellular senescence via its binding proteins, we employed biotin‐labelled DNA probes (Figure S1A) to enrich NEAT1 and its binding protein from control and serum starvation‐induced senescent HepG2 cell lysates. NEAT1‐binding proteins were identified using protein mass spectrometry (Figure 5A). The results showed that NEAT1 was enriched in paraspeckle‐associated proteins (NONO, PSPC1 and SFPQ) and KIF11. Serum starvation weakened the ability of NEAT1 to bind paraspeckle‐associated proteins. Interestingly, the levels of KIF11 were significantly increased in NEAT1‐enriched lysate under serum starvation (Figure 5A). This change was further validated in subsequent RNA pulldown experiments (Figures 5B and C). We then detected the changes of KIF11 in senescent hepatoma cells. We found that ROS stress led to the down‐regulation of KIF11 protein level, and this was reversed when cells were treated with MG132 (Figure 5D). Furthermore, the protein half‐life assay showed that serum starvation drastically shortened the half‐life of KIF11 from the normal 1 h to less than 20 min (Figure 5E). These results suggest KIF11 protein degradation in senescent cells.

ROS accumulation extensively promotes protein degradation in the cytoplasm, which is one of the main reasons for the decreased level of KIF11 protein. Next, we explored whether the degradation of KIF11 protein is due to its interaction with NEAT1 in cytoplasm of senescent cells. As ROS stress induced senescence of hepatoma cells and increased NEAT1 expression in the cytoplasm (Figures 4E and S3A, B). We then constructed doxycycline (DOX)‐induced NEAT1‐knockdown HepG2 cells (Figure 5F) to instantaneously reduce NEAT1 levels in both cytoplasm (Figure 5G) and nucleus (Figure 5H). We further found that the half‐life of KIF11 in DOX‐induced NEAT1‐knockdown HepG2 cells was not shortened compared with control cells, cultured not only in complete medium but also in serum‐free medium (Figures S1A and 5I). We performed NEAT1‐FISH and KIF11 IF in control and senescent HepG2 and HCCLM3 cells and found that NEAT1 was translocated from the nucleus to the cytoplasm to be co‐localised with KIF11 under serum starvation conditions (Figure 5J). FISH‐IF assays also displayed that both NEAT1 and KIF11 were reduced under serum starvation (Figure 5J). Paraspeckles serve as a ware house for many proteins and RNAs and control of gene expression through mRNA storage.^39^, ^40^ Our previous study has also published that paraspeckle disassembly in activated macrophages resulted in the nuclear export of NEAT1 and degradation of its component molecules, including proteins and RNAs^38^. Here, we further detected the half‐life of NEAT1 in control or ROS stress‐induced HepG2 cells. As shown in Figure S3C, ROS stresses resulted in a decrease of the half‐life of NEAT1 in HepG2 cells from 8 to 4 h. It is indicated that the stability of NEAT1 in cancer cells is weakened by ROS stresses, which is one of the reasons for the decreased NEAT1 expression in senescent cancer cells. These results demonstrate that the degradation of KIF11 protein is dependent on its interaction with NEAT1 in cytoplasm.

In addition, ROS stress led to mRNA down‐regulation of KIF11, and ROS scavenger Tempol rescued this change to a certain extent (Figure S3D). To further assess mRNA down‐regulation of KIF11 in senescent hepatoma cells, we cloned the KIF11 promoter region (2 kb upstream of the 5' end of gene) into a transcriptionally active luciferase reporter plasmid. The cells were then transiently transferred into HepG2 cells to construct short‐acting KIF11 transcriptional activity reporter cells. The results presented that ROS stress led to the down‐regulation of transcription activity in the KIF11 promoter region, and Tempol rescued this change (Figure S3E). Paraspeckle‐associated proteins play vital roles in the regulation of gene transcription and expression in the nucleus.^41^, ^42^, ^43^ Similarly, knockdown of NONO and PSPC1 resulted in intracellular paraspeckle depolymerisation and inhibited transcription activity in the KIF11 promoter region (Figures S3E–G). Altogether, under ROS stress, paraspeckle depolymerisation down‐regulates mRNA level of KIF11 and NEAT1 translocation into the cytoplasm, which binds to KIF11 protein and promotes protein degradation of the latter (Figure 5K).

### 2.5 KIF11 negatively correlates with senescence biomarkers in HCC tissues and hepatoma cells

**FIGURE 6:**
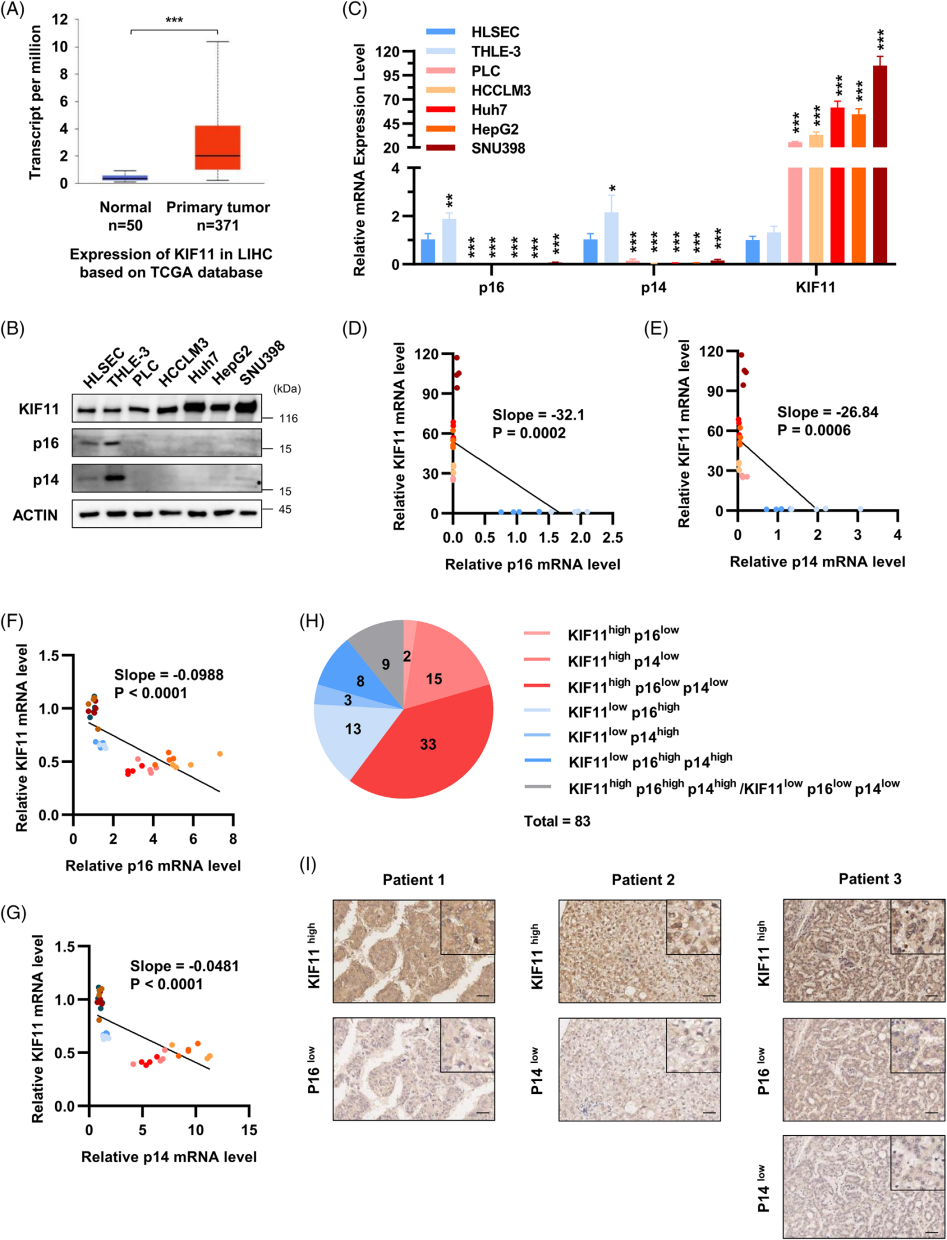
Expression level of KIF11 and CDKN2A (p16 and p14) in HCC. (A) Based on the mRNA expression profile data and clinical information in the TCGA database, KIF11 expression in HCC tissues and associated adjacent tissues were displayed in boxplots. Protein (B) and mRNA (C) level of KIF11, p16 and p14 in cultured normal liver cells (HLSEC and THLE‐3) and hepatoma cells (HCCLM3, Huh7, HepG2, SNU398). (D) Correlation analysis of KIF11 and p16 in cultured normal liver cells (HLSEC and THLE‐3) and hepatoma cells (HCCLM3, Huh7, HepG2, SNU398). (E) Correlation analysis of KIF11 and p14 in cultured normal liver cells (HLSEC and THLE‐3) and hepatoma cells (HCCLM3, Huh7, HepG2, SNU398). (F) Correlation analysis of KIF11 and p16 in ROS stress‐induced normal liver cells (THLE‐3) and hepatoma cells (Huh7 and HepG2). (G) Correlation analysis of KIF11 and p14 in ROS stress‐induced normal liver cells (THLE‐3) and hepatoma cells (Huh7 and HepG2). (H) Pie chart of different expression levels of KIF11, p16 and p14 in clinical HCC tissues. A total of 83 clinical cases were analysed. 2 cases were KIF11^high^ p16^low^, 15 cases were KIF11^high^ p14^low^, 33 cases were KIF11^high^ p16^low^ p14^low^, 13 cases were KIF11^low^ p16^high^, 3 cases were KIF11^low^ p14^high^, 8 cases were KIF11^low^ p16^high^ p14^high^ and 9 cases were KIF11^high^ p16^high^ p14^high^ /KIF11^low^ p16^low^ p14^low^. (I) IHC pictures of KIF11^high^ p16^low^ expression in patient 1, IHC pictures of KIF11^high^ p14^low^ expression in patient 2 and KIF11^high^ p16^low^ p14^low^ expression in patient 3. The scale bar indicates 50 μm. Data shown are the mean ± SD (*n* ≥ 3; *p < .05, **p < .01, ***p < .001, two‐tailed *t*‐test).

KIF11, also known as Kinesin‐5, is a necessary molecular motor protein during mitosis.^44^, ^45^ KIF11 mediates centromeric separation and bipolar mitotic spindle formation, thereby promoting mitosis to support cell proliferation.^46^ KIF11 is highly expressed in various malignancies,^47^, ^48^ including HCC.^49^, ^50^ The role of KIF11 in modulating cellular senescence has not been reported. We re‐analysed the TCGA database and GTEx database and found that KIF11 was highly expressed in HCC tissues compared with their adjacent normal tissues (Figure 6A). Likewise, KIF11 was highly expressed in cultured hepatoma cells (HCCLM3, Huh7, HepG2 and SNU398) compared with normal liver cells (HLSEC and THLE‐3) (Figures 6B and C). Furthermore, KIF11 is negatively corelated with p16 and p14 in cultured liver cells and hepatoma cells (Figures 6D and E). These effects were also observed in ROS stress‐induced cultured normal liver cells (THLE‐3) and hepatoma cells (HepG2 and Huh7) (Figures S4A and 6F, G).

We then analysed the correlation among the KIF11, p16 and p14 protein levels in clinical HCC tissues. A total of 83 cases of HCC tissues were stained by IHC and scored according to staining intensity. As shown in the pie chart (Figures 6H and S4B, C), 2 cases were KIF11^high^ p16^low^, 15 cases were KIF11^high^ p14^low^, 33 cases were KIF11^high^ p16^low^ p14^low^. IHC pictures showed different expression of KIF11, p16 and p14 in clinical HCC tissues (Figures 6I and S4D). Totally, in about 60% (50 in 83 cases) of HCC patients, the expression of KIF11 is higher, while p16 or (and) p14 is low. These results indicate that the high expression of KIF11 inhibits the cellular senescence in the liver of patients with HCC. Altogether, KIF11 negatively correlates with senescence in both clinical HCC tissues and cultured hepatoma cells.

### 2.6 KIF11 knockdown leads to cellular senescence and restrains HCC progression

**FIGURE 7:**
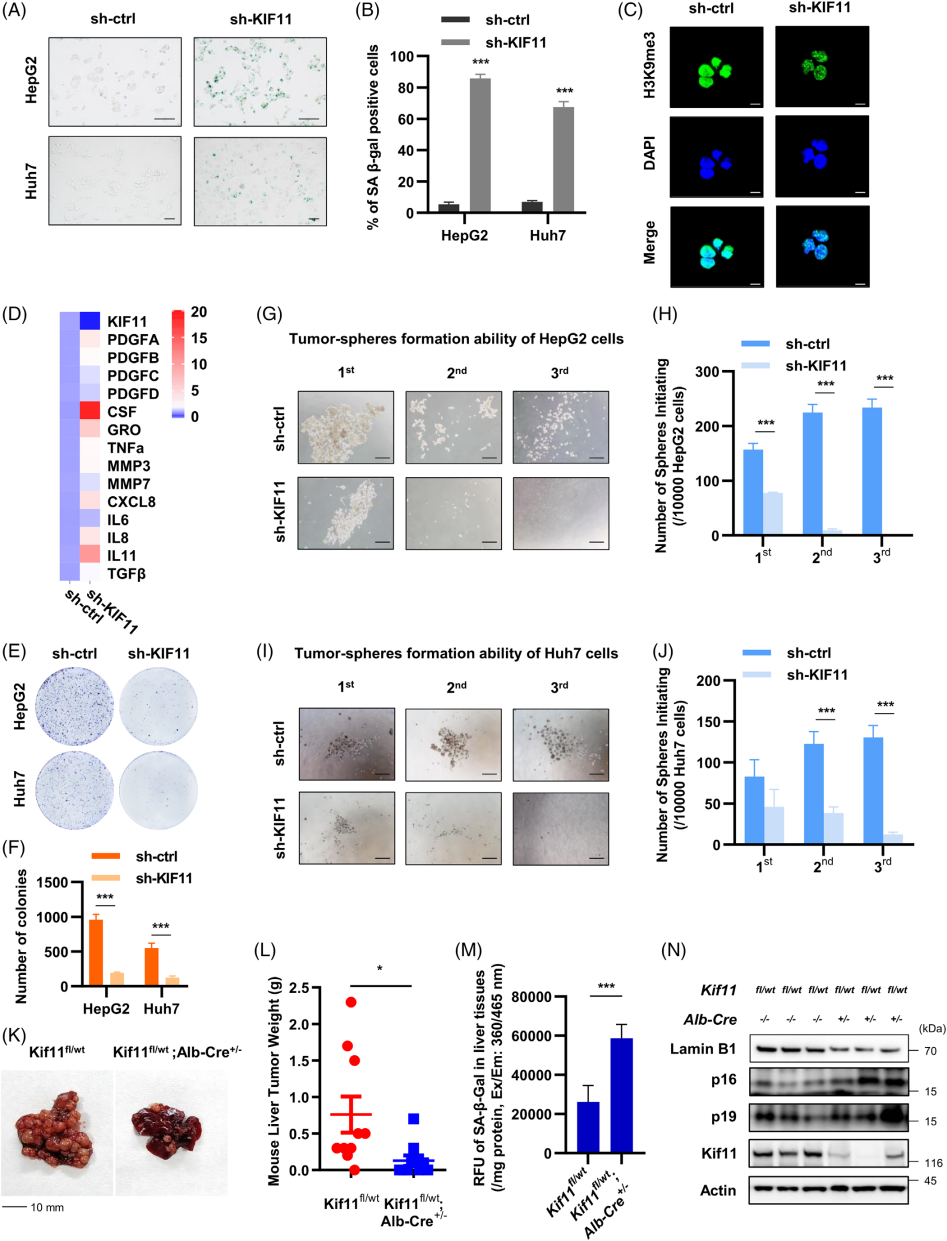
Effect of KIF11 on cellular senescence and tumour growth of HCC. SA‐β‐galactosidase staining (A and B) and heterochromatin foci formation (C) were detected in sh‐ctrl or sh‐KIF11 HepG2 and Huh7 cells. In A, the scale bar indicates 100 μm. In C, the scale bar indicates 10 μm. (D) SASP in sh‐ctrl or sh‐KIF11 HepG2 cells was detected and shown by qPCR. The average of relative expression levels were shown in the heatmap (*n* = 3). (E and F) Clone formation assay was used to detect the clone formation ability of sh‐ctrl or sh‐KIF11 HepG2 and Huh7 cells. The data were presented in column graph. (G–J) Tumour‐spheres formation assay was used to detect the self‐renewal ability of sh‐ctrl or sh‐KIF11 HepG2 and Huh7 cells. The scale bar indicates 500 μm. (K–M) Liver tumour (K), tumour weight (L) and liver SA‐β‐Gal activity (M) of *Kif11^fl/wt^* and *Kif11^fl/wt^*;*Alb‐Cre^+/−^* mouse, each group contains 10 mice. (N) Protein levels of Kif11, p16, p19 and Lamin B1 in liver tumour from *Kif11^fl/wt^* and *Kif11^fl/wt^*;*Alb‐Cre^+/−^* mouse. Data shown are the mean ± SD (*n* = 10; *p < .05, **p < .01, ***p < .001, two‐tailed *t*‐test).

To explore the effect of KIF11 on cellular senescence in hepatoma cells, lentiviral system was used to stably knockdown or overexpress KIF11 in HepG2 and Huh7 cells. We found that KIF11 deficiency increased the proportion of SA‐β‐Gal positive hepatoma cells (Figures 7A and B) and the formation of heterochromatin foci in the nucleus of HepG2 cells (Figure 7C). In contrast, KIF11 overexpression reduced senescent phenotype caused by serum starvation (Figures S2A–C). Also, we found that knocking down KIF11 activated SASP, expression level of PDGF family, cytokines (CSF, CRO, TNF‐α, TGF‐β, IL‐6, IL‐8, IL‐11) and chemokines (CXCL8) were up‐regulated (Figure 7D). KIF11 overexpression reduced SASP caused by serum starvation (Figure S2D). Besides, we used KIF11 inhibitors SB743921 and Ispinesib, which can inhibit proliferation and induce apoptosis, to treat HepG2 cells and found they inhibited proliferation of HepG2 cells (Figures S5A and B). SB743921 selectively binds the ATP‐binding domain on kinesin spindle protein (KSP), blocks mitotic spindle assembly.^46^ Ispinesib alters the binding of KSP to microtubules and inhibits KSP movement by blocking the release of ADP.^46^, ^51^ Interestingly, there was no difference in SA‐β‐Gal positive hepatoma cells between DMSO and SB743921 or ispinesib‐treated group (Figures S5C and D). Neither SB743921 nor Ispinesib affect senescence biomarkers (Figure S5E). These data suggest that the effect of KIF11 on cellular senescence is independent of its canonical molecular motor function. The regulation of KIF11 in cell senescence involves other signalling pathways. More importantly, through rescue experiments, it was found that KIF11 overexpression reversed the senescence phenotype in hepatoma cells caused by NEAT1 deletion (Figures S6A–C). This indicates that the effect of NEAT1 on hepatoma cell senescence is dependent on KIF11.

Next, we studied the effect of KIF11 on HCC development. First, in vitro experiments show that knockdown KIF11 inhibited clone formation (Figures 7E and F) and tumour‐spheres formation of hepatoma cells (Figures 7G–J). We then used liver‐specific Kif11 knockout (*Kif11^fl/wt^*;*Alb‐Cre^+/−^*) mice and WT (*Kif11^fl/wt^*) littermates to determine their tumorigenesis in a mouse model of HCC. We found that the malignant degree (Figure 7K) and weight (Figure 7L) of liver tumours in *Kif11^fl/wt^*;*Alb‐Cre^+/−^* group were lower than those in the WT mice.  Furthermore, SA‐β‐Gal activity, p16 and p19 (p14 in human) were up‐regulated in liver cancer tissues of the *Kif11^fl/wt^*;*Alb‐Cre^+/−^* mice compared with the *Kif11^fl/wt^* mice (Figures 7M and N). Additionally, as shown in Figure 7N, the liver tissues of the *Kif11^fl/wt^*;*Alb‐Cre^+/−^* mice showed loss of Lamin B1, compared with those of *Kif11^fl/wt^* littermates. Thus, knockdown of KIF11 activates the p16 and p14 signalling pathways and inhibits the development of HCC.

To furtherly identity the function of cellular senescence in HCC progression, which has been shown in Figures 3 and 7, various hepatoma cells (HepG2, HCCLM3 and Huh7) were treated with two cellular senescence inhibitors (Tempol and JAKi).^52^, ^53^, ^54^, ^55^ and subjected to colony formation assay. As shown in Figures S6E and F, cellular senescence inhibitors could promote colony formation of hepatoma cells cultured with or without serum, which indicates that cellular senescence inhibit HCC progression.

### 2.7 KIF11 maintains the stability of WNT6, WNT7B and WNT8B, which inhibits transcriptional activation of CDKN2A in hepatoma cells

**FIGURE 8:**
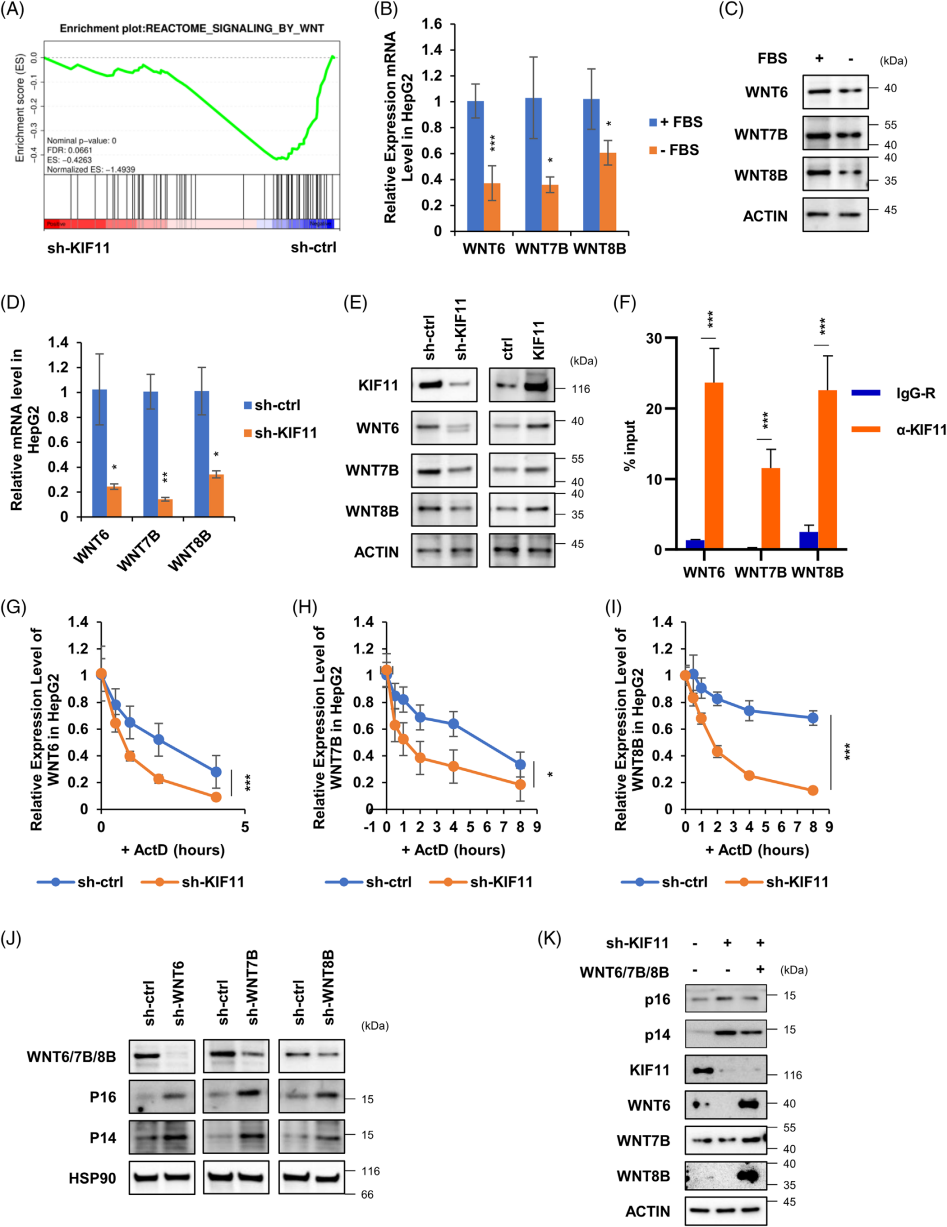
KIF11 maintains the mRNA stability of WNT6, WNT7B and WNT8B. (A) Control and KIF11 knockdown HepG2 cells were used for mRNA sequencing. The expression profile was used for GSEA on the WNT signalling pathway. HepG2 cells were cultured in a serum‐free medium for 0 or 48 h, mRNA expression levels of WNTs were analysed by qPCR (B), while protein expression levels were detected by WB (C). Control and KIF11 knockdown or overexpression HepG2 cells were used for qPCR (D) and WB (E) analysis. (F) Cell lysates of HepG2 cells were incubated with normal rabbit IgG or KIF11 antibody for RIP. The immunoprecipitates were analysed by real‐time RT‐PCR to examine the enrichment efficiency of WNTs mRNA. (G–I) HepG2 cells expressing control shRNA or KIF11 shRNA were treated with actinomycin D (1 mg/mL) for the indicated periods. Total RNA was then analysed by real‐time RT‐PCR to examine the mRNA half‐life of indicated mRNAs. (J) HepG2 cells were infected with lentiviruses expressing sh‐ctrl, sh‐WNT6, sh‐WNT7B or sh‐WNT8B. The protein lysates were subjected to WB analysis. (K) sh‐ctrl&ctrl, sh‐KIF11&ctrl or sh‐KIF11&WNT6/7B/8B HepG2 cells were used for WB analysis with indicated antibodies. Data shown are the mean ± SD (*n* ≥ 3; *p < .05, **p < .01, ***p < .001, two‐tailed *t*‐test).

To investigate the molecular mechanism of KIF11 in repressing cellular senescence, we conducted Gene Set Enrichment Analysis (GSEA) on cells in the control and KIF11‐knockdown cells based on transcriptome sequencing. We found a positive correlation between KIF11‐knockdown and the molecular changes in the WNT signalling pathway (Figure 8A). Activation of the WNT signalling pathway is related to the occurrence and development of various cancers,^56^, ^57^ while the reduced WNT signalling is associated with aging and Alzheimer's disease.^58^ There are 19 WNT proteins in humans, and their homology is extremely high, and WNT signalling declined in senescent cells in many tissues with age, such as the liver, brain, skeletal muscle and lung.^59^ We analysed the mRNA levels of the above identified WNT genes in HepG2 cells cultured in normal or serum‐free conditions. WNT6, WNT7B and WNT8B gene expression was all decreased in serum starved HepG2 cells, whereas the other WNTs mRNA shown no significant changes (Figure 8B, the additional data not shown). Additionally, the pre‐mRNA levels of WNT6, WNT7B and WNT8B did not change (Figure S7A), suggesting that WNT6, WNT7B and WNT8B mRNAs were post‐transcriptionally regulated by serum starvation. Similarly, the protein levels of WNT6, WNT7B and WNT8B decreased in serum starved HepG2 cells (Figure 8C). This phenotype was also found in the KIF11 knockdown (sh‐KIF11) HepG2 cells (Figures 8D, E and Figure S7B). In contrast, WNT6, WNT7B and WNT8B protein levels were increased in KIF11 overexpressed HepG2 cells (Figure 8E). Furthermore, KIF11 interacted with mature WNT6, WNT7B and WNT8B mRNAs (Figures 8F and S7C). Finally, KIF11 deficiency enhanced the degradation of WNT6, WNT7B and WNT8B mRNAs (Figures 8G–I). Altogether, these results demonstrate that KIF11 maintains WNTs mRNA stabilisation.

WNT6, WNT7B and WNT8B are oncogenes that play critical roles in canonical WNT signal pathway.^60^ WNTs activate β‐catenin, which transcriptionally represses CDKN2A expression.^61^, ^62^ We found the deficiency of WNT6, WNT7B and WNT8B transcriptionally activated p16 and p14 in HepG2 cells (Figures 8J and S7D–F). However, overexpression of WNT6/7B/8B did not totally rescue p16/p14 senescence pathways caused by knockdown of KIF11 (Figure 8K). This indicates that there are other signalling pathways involve in cellular senescence process of HCC caused by KIF11 down‐regulation. Altogether, serum starvation‐induced ROS stress decrease the expression of KIF11, as well as WNT6, WNT7B and WNT8B, and this transcriptionally activates CDKN2A.

### 2.8 KIF11‐H3.3‐TET2 axis represses DNA demethylation of CDKN2A in hepatoma cells

**FIGURE 9:**
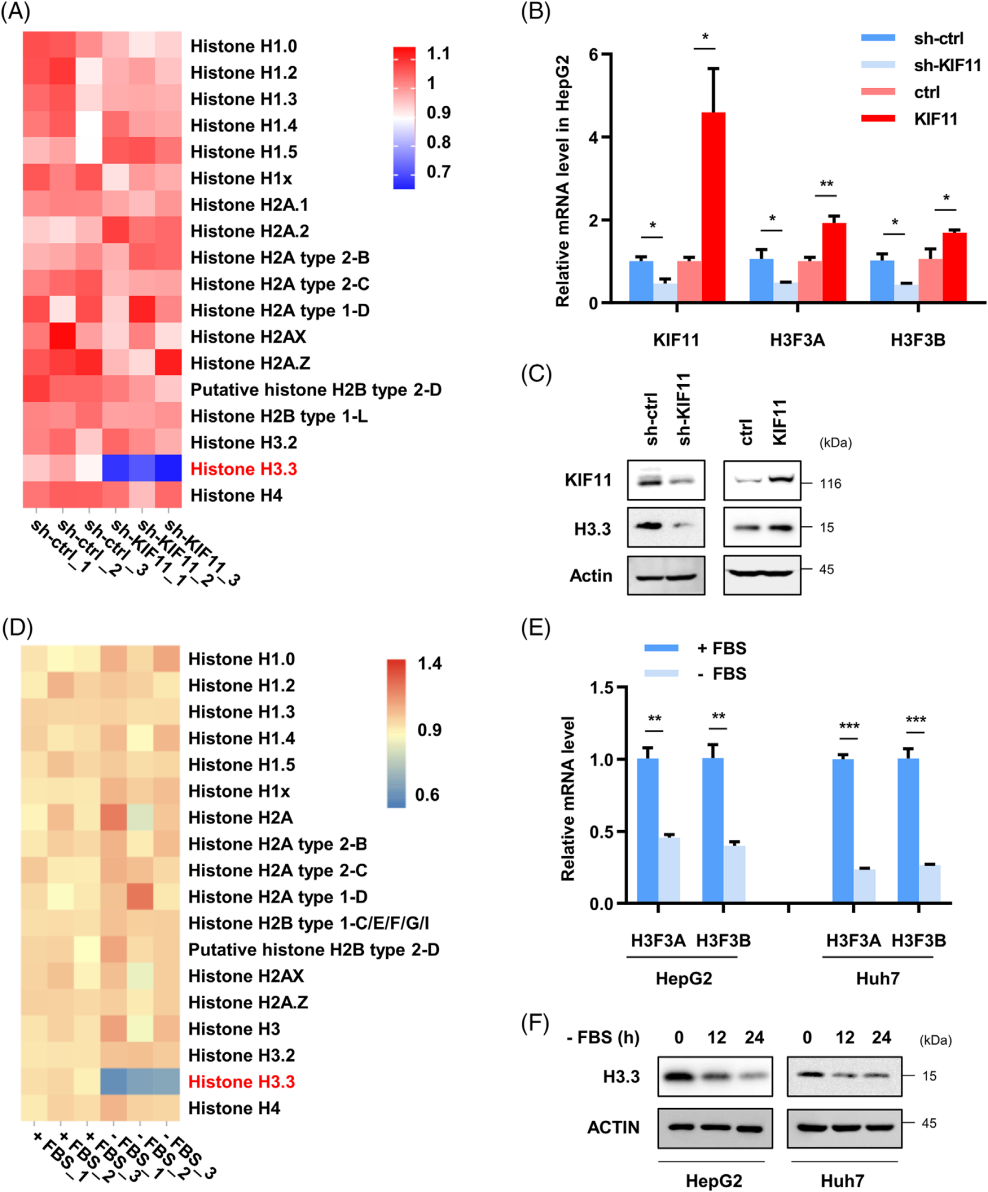
sh‐KIF11 or serum starvation down‐regulates the expression of H3.3. Heatmap view of TMT‐based quantitative proteomics analysis of main histone proteins on sh‐ctrl and sh‐KIF11 cells. A lentiviral system was used to knockout or overexpress KIF11 stably in HepG2 cells. mRNA (B) and protein (C) levels of H3.3 were detected. (D) Heatmap view of TMT‐based quantitative proteomics analysis of main histone proteins on + FBS or −FBS‐treated hepatoma cells. (E and F) mRNA (E) and protein (F) levels of H3.3 in +FBS or −FBS‐treated hepatoma cells were detected. Data shown are the mean ± SD (*n* ≥ 3; *p < .05, **p < .01, ***p < .001, two‐tailed *t*‐test).

To further explore other potential signalling pathways involved in KIF11 regulated cellular senescence, we conducted proteomic analysis on control and KIF11‐knockdown HepG2 cells. The changes in histones brought our attention. There are five variants of histone H3, including classical H3.1 (mammalian specific expression) and H3.2, alternative variant H3.3, centromere‐specific CenH3, and testicular specific H3t.^63^ H3.3 is the only histone expressed throughout the cell cycle, and targets active transcriptional sites throughout the cell cycle.^64^ As shown in the heatmap, histone H3.3 was decreased significantly in sh‐KIF11 HepG2 cells (Figure 9A). Human H3.3 is encoded and translated by two independent genes, H3F3A and H3F3B. We next confirmed that KIF11 positively regulated H3.3 at not only mRNA (Figure 9B) but also protein (Figure 9C) levels in HepG2 cells. Serum starvation causes oxidative stress by increased ROS levels.^65^ Increasing ROS could result in DNA damage.^66^ Thus, serum starvation could be used as a method to induce senescence via different pathways including ROS and DNA damage. Serum starvation, ROS and DNA damage are synergistic in the process of cellular senescence, and the relationship is complex.^67^, ^68^ In this study, we used serum starvation to induce senescence. Based on proteomic analysis, Histone H3.3 was also decreased in serum starved HepG2 cells (Figure 9D). H3.3 was down‐regulated at both mRNA (Figure 9E) and protein (Figure 9F) level in serum starved HepG2 and Huh7 cells.

**FIGURE 10:**
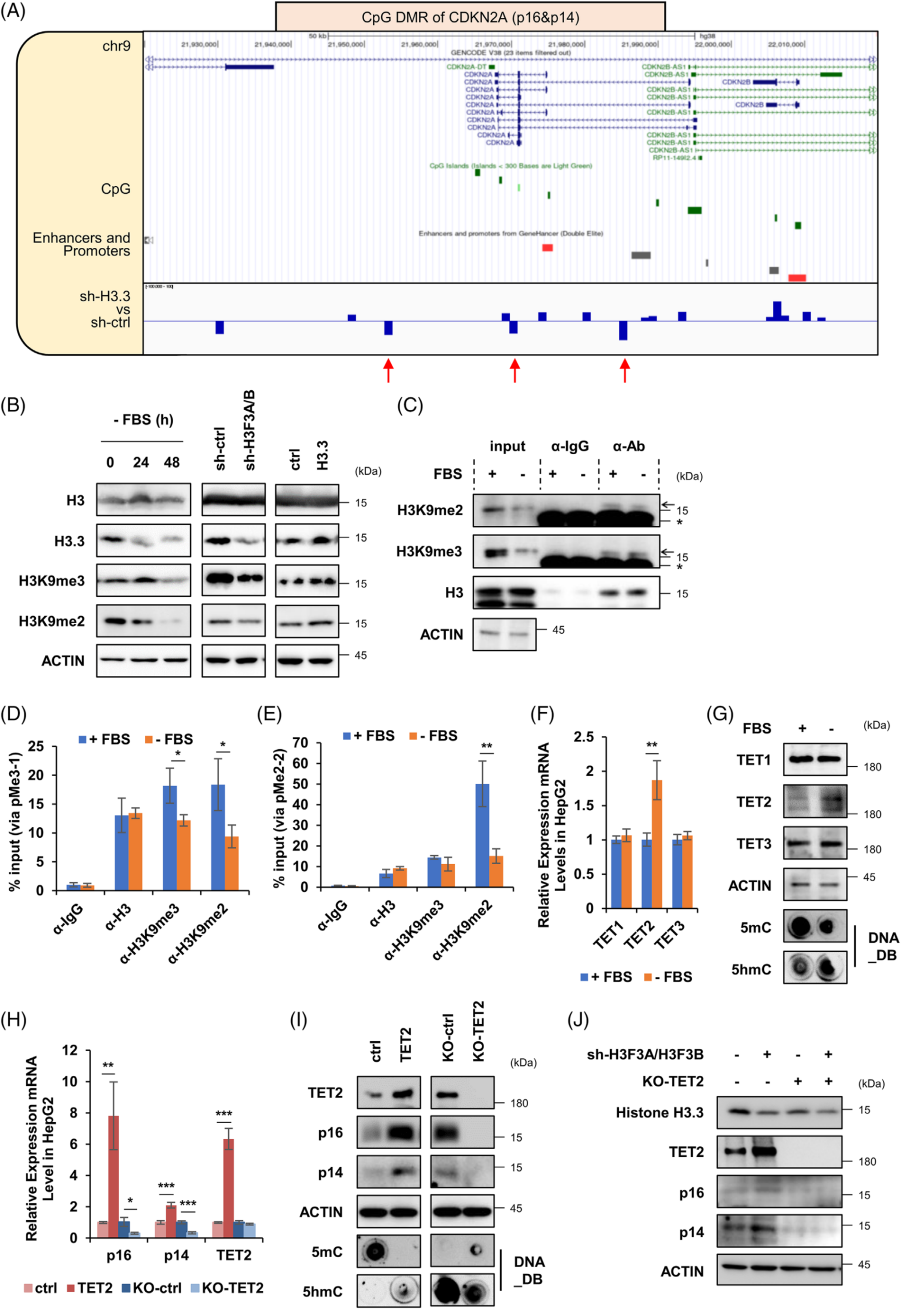
H3.3 down‐regulation leads to demethylation of CDKN2A through methyltransferase TET2. (A) Schematic illustration of gene location and CpG island distribution of CDKN2A in control and H3.3‐knockdown HepG2 cells, changes in genomic DNA modification. (B) Control, serum‐starved, H3.3 knockdown, H3.3 overexpression HepG2 cells were used to detect various modification types of histone H3 by WB with indicated antibodies. Control and serum‐starved HepG2 cells were used for ChIP analysis via indicated DNA modification antibodies. And the enriched protein and genomic DNA segments were analysed by WB (C) and qPCR (D and E). mRNA (F) and protein (G) levels of the TET protein family (TET1, TET2 and TET3) were analysed in control or serum‐starved HepG2 cells. A lentiviral system was used to knockout or overexpress TET2 stably in HepG2 cells. mRNA (H), protein and DNA methylation (I) levels in cellular senescence‐related markers were detected. (J) sh‐ctrl&KO‐NTC, sh‐H3.3&KO‐NTC, sh‐ctrl&KO‐TET2 or sh‐H3.3&KO‐TET2 HepG2 cells were used for WB analysis with indicated antibodies. Data shown are the mean ± SD (*n* ≥ 3; *p < .05, **p < .01, ***p < .001, two‐tailed *t*‐test).

Therewith, we tested the effect of serum starvation, KIF11 and H3.3 on the mRNA expression level of CDKN2A genes. As expectations, mRNA levels of p16 and p14 were higher in serum‐starved, KIF11‐deficient or H3.3‐deficient HepG2 cells, compared with corresponding control cells (Figure S8A). In contrast, not only KIF11 but also H3.3 overexpression inhibited the mRNA expression of p16 and p14 (Figure S8A). DNA methylation of CpG island, mapping in the promoter region of a gene, would inhibit gene expression by recruiting transcription repressors or hindering the binding of transcription factors.^69^ Changes in the methylation of DNA or histones induce epigenetic changes that contribute to aging and cancer development.^29^, ^70^, ^71^ We performed KEGG pathway analysis in genes within differentially methylated regions and differentially methylated promoters (DMPs). We found that H3.3‐epigenetically regulated genes enriched in aging, cell growth and death pathways (Figures S8B and C). As shown on the UCSC Genome bioinformatics website, several CpG islands located in the promoter regions of CDKN2A (p16 and p14) (Figure 10A). This indicates that DNA methylation has the potential to alter the expression of CDKN2A. DNA methylation is crucial in understanding how H3.3 regulates the transcriptional expression of CDKN2A. Thus, we preformed whole‐genome bisulfite sequencing (WGBS) and analysis of the control and H3.3 knockdown cells. Methylation of CpG sites in the promoter and enhancer regions of CDKN2A genes was decreased in H3.3 knockdown cells compared with control cells (Figure 10A). Meanwhile, inhibition of H3.3 expression did not cause genome‐wide DNA methylation differences in HepG2 cells (Figures S8D and E), suggesting that H3.3 specifically regulates CDKN2A gene methylation.

Histone H3 is the most modified histone in cancer.^72^ The most common modifications are methylation, acetylation and phosphorylation. Therefore, we tested the effects of various treatments such as serum starvation, knockdown of H3.3 and overexpression of H3.3 on the overall histone modification level in HepG2 cells. We found that serum starvation or knockdown of H3.3 down‐regulated H3K9me3/me2 levels (Figure 10B). In contrast, the levels of these modifications were up‐regulated by H3.3 overexpression (Figure 10B). H3K9me3 and H3K9me2 are the critical repressive marks locating round gene body, especially promoter regions.^73^ According to the H3K9me3 and H3K9me2 chromatin immunoprecipitation (ChIP)‐seq peaks in ENCODE database,^74^ we found the potential histone modification DNA regions around the TET2 gene (Figures S8F and G). Moreover, the subsequent ChIP assay (Figure 10C) and qPCR analysis (Figures 10D, E and S8H‐J) showed that TET2 gene region contained lower levels of associated modified H3 (H3K9me3 and H3K9me2) in serum‐ starved HepG2 cells. These results suggest that under serum starvation condition, histone modifications in the TET2 gene region are reduced, and thus H3.3 expression is decreased.

Ten eleven translocation (TET) enzymes family, including TET1, TET2 and TET3.^75^ TET enzymes catalyse 5‐methylcytosine (5mc) to 5‐hydroxymethylcytosine (5hmc), demethylate DNA^75^ and regulate gene transcription.^76^ Recently study found that TET2 affects CDKN2A methylation and expression.^77^ We found that TET2 expression was up‐regulated in serum‐starved hepatoma cells, while the expression of TET1 and TET3 did not change (Figures 10F and G). Meanwhile, DNA Dot Blot showed that 5mc was down‐regulated and 5hmc was up‐regulated (Figure 10G). Further, TET2 overexpression up‐regulated p16 and p14 at both mRNA and protein levels, while knock‐off TET2 showed an opposite effect (Figures 10H and I). In addition, H3.3 deficiency significantly increased the protein level of TET2 (Figure 10J) and activated p16 and p14 in control (KO‐NTC) HepG2 cells, but not in TET2 knockout (KO‐TET2) cells (Figure 10J). This rescue experiment proved that the regulation of H3.3 on CDKN2A gene is dependent on TET2.

Altogether, KIF11‐H3.3‐TET2 axis represses DNA demethylation of CDKN2A in HCC. Under ROS stress condition, KIF11‐H3.3 down‐regulation leads to demethylation of CDKN2A through TET2 to induce cellular senescence in HCC.

## 3 DISCUSSION

NEAT1 has been reported highly expressed and functions as an oncogene in several cancers including prostate cancer,^78^ laryngeal squamous cell carcinoma,^79^, ^80^ breast cancer,^81^, ^82^ ovarian cancer,^83^ colorectal cancer,^84^ gastric cancer,^85^ glioma^86^ and oesophageal squamous cell carcinoma.^87^ In HCC, overexpression of NEAT1 promotes tumour progression and metastasis,^88^, ^89^ and maintains the properties of cancer stem cells.^90^ Here, we showed that NEAT1 is overexpressed in both HCC tissues and hepatoma cells, and further established its negative correlation with senescence in HCC. These findings also demonstrated that NEAT1 is a pan‐cancer LncRNA and may serve as an indicator of tumour prognosis.

It has been reported that in cancer development, lncRNA NEAT1 binds to miRNAs as a competing endogenous RNA (ceRNA), thus affects the expression levels of their target genes. Wang and coworkers^91^ summarised roles of NEAT1/miRNA/target axis in the progression of various cancers. Further findings on upstream regulatory proteins of NEAT1 demonstrates that p53 induces NEAT1 expression and paraspeckle formation.^92^ In HCC, prior reports propose that NEAT1 promotes tumour progression and metastasis via regulating miR‐5129‐5p/VCP/IκB axis,^93^ miR‐139‐5p/TGF‐β1 axis^94^ or miR‐485/STAT3 axis.^95^ In the present study, we provide new insights that NEAT1 promotes HCC by inhibiting senescence via KIF11‐dependent repression of CDKN2A.

NEAT1 is localised in paraspeckles,^96^ while external stress causes changes in NEAT1 expression and localisation. For example, glucose stimulates Pinin loading onto NEAT1 and enhances its translocation to the cytoplasm.^82^ Under inflammasome‐activating stimuli, Neat1 translocates to the cytoplasm after release from paraspeckles.^38^ Furthermore, NEAT1 is down‐regulated in both replicative senescent fibroblasts cells (GSE77675 and GSE116761 datasets) and ROS‐treated senescent cells (GSE116761 and GSE144510 dataset). Replicative senescence in cultured hepatocytes and cirrhosis is associated with reduced telomerase activity.^97^, ^98^ In contrast, hepatoma cells exhibit p53 inactivation mutations and epigenetic silencing of p16^INK4^.^99^ ROS stress induces cellular senescence through activation of different signalling pathways.^28^ Here, as shown in Figure S9, during ROS stress‐induced cellular senescence in hepatoma cells, NEAT1 translocates to the cytosol, which interacts and leads to KIF11 degradation. KIF11 down‐regulation leads to transcriptional activation and DNA demethylation of CDKN2A. This activates the p16 and p14 signalling pathways and drives the senescence of hepatoma cells. Cellular senescence thus led to growth arrest and inhibit the progression of hepatoma cells. This function of NEAT1 on cellular senescence is paraspeckle‐independent. Our findings also revealed new mechanisms of NEAT1 down‐regulation in suppressing HCC progression. Furthermore, we need to discuss that aging and cellular senescence are different concepts. Aging is a progressive decline with time. However, senescence occurs since embryogenesis and throughout the lifespan.^100^ In addition to acute cellular senescence, other factors, including mitochondrial dysfunction and telomere shortening, also contribute to aging.^101^, ^102^ In the present study, we focused on mechanisms underlying cellular senescence rather than aging during the development of HCC.

At present, inducing cellular senescence has been used to treat cancer practice.^9^, ^103^ However, cellular senescence has double‐sided effect in tumours mainly due to different role of SASP.^9^ SASP have both positive (immune surveillance) and negative (immunosuppressive) effects on senescent cells in tumors.^9^ This dual effect of SASP dependent on tumour microenvironment during the different stages of cancer progression.^104^ Senescence can also potentiate oncogenesis. Senescent cells induced ectodomain shedding of E‐cadherin, thus promotes lung metastasis.^105^ Cellular senescence in malignant cells often promotes adverse effects of chemotherapy.^106^ Therapy‐induced senescence can also cause cancer metastasis and relapse.^107^ Therefore, inducing senescence of cancer cells earlier and selective elimination of these senescent cancer cells later would achieve the effect of radical tumour treatment. In 2015, Dr. James Kirkland's team discovered drugs that selectively kill senescent cells called senolytics.^108^ Small molecule senolytic drugs are promising strategies for cancer prevention and treatment in clinic.^109^ The concept of 'one‐two punch' cancer therapy, therapeutics to promote senescence of tumour cell followed by selective clearance, has been coming up to improve cancer patients’ treatment outcomes.^13^ Inducing cell senescence in the early stages of tumour development can inhibit the rapid progression of tumours, thus preparing for later radical therapies and prolonging patients' survival. The effectiveness of the 'one‐two punch' therapy has been verified in a variety of mouse models.^12^ Nevertheless, the current 'one‐two punch' therapy is still at the level of experimental animals, and further preclinical trial are needed to determine whether it can achieve clinical efficacy in inhibiting the development of HCC.

At last, our findings indicate potential clinical implications. Based on our discovery, NEAT1 and KIF11 are overexpressed in HCC tissues and hepatoma cells, and both are negatively correlate with senescence. Some kinesin proteins are related to cancer malignancy and drug resistance, which would be anticancer targets.^110^ Especially, inhibitors of KSP (KIF11/Eg5) have entered clinical trials for monotherapy or in combination with other drugs.^111^, ^112^, ^113^, ^114^ NEAT1 is associated with cancer initiation, metastasis, recurrence and patient survival.^115^ However, the clinical trial for cancer treatment, based on NEAT1 studies, has not been reported. Thus, the combination treatment strategy for cancers, especial HCC, by inhibiting NEAT1 and KIF11, has great potential for clinical application. Furthermore, it seems that senescence markers may also serve as a diagnosis or prognostic biomarker of HCC. High expression of senescence markers in tumour cells indicates tumour cell growth arrest, which may indicate a good prognosis. The long‐term presence of senescent cells indicates drug resistance or recurrence of tumour cells.^116^ Thus, eliminating these senescent cells would achieve the goal of radical tumour treatment.^12^ Nevertheless, for rapidly progressing tumours, such as HCC, early control of malignant progression of tumours is still of great clinical significance to provide more time for later radical treatment and prolong the survival of patients. Knockdown NEAT1 or KIF11, serum starvation, H_2_O_2_ or DOXO treatment are the ways to induce senescence in hepatoma cells. Our discovery of the role of NEAT1, KIF11 and senescence in HCC therefore provides new idea for therapy opportunities. Still, our study has some limitations. For example, all our findings have only been validated in cells and mouse models. In the future, the inhibitory effect of inducing cell senescence on the development of HCC should be further investigated using organoid models or PDX models. In the end, how to eliminate these senescent cells at a later stage to achieve the goal of radical treatment of HCC needs more research. In conclusion, NEAT1 suppresses cellular senescence in HCC via KIF11‐dependent repression of CDKN2A. High expression of NEAT1 or KIF11 inhibits hepatocellular senescence in clinical HCC and cultured hepatoma cells. Targeting NEAT1 or KIF11 to induce hepatocellular senescence is a potential therapy to restrain HCC development.

## 4 METHODS

### 4.1 Cellular senescence induction

Various oxidative stresses, including exposure to serum‐deprived medium (48 h), H_2_O_2_ (100 μM, 24 h) and DOXO (100 nM, 24 h), were performed to induce premature senescence.

### 4.2 Senescence β‐galactosidase activity assay

The senescence β‐galactosidase staining kit (Beyotime; RG0039) was used to mark senescent cells. The control and experimental group cells were fixed in stationary liquid. After 20 min, cells were dyed in staining reagent for 8–12 h at 37°C. Pictures were taken by a microscope (Olympus).

### 4.3 Immunofluorescence assay

H3K9me3 immunofluorescence analyses were carried out to analyse the heterochromatin foci formation of senescent cells. NONO and PSPC1 immunofluorescence staining analyses were carried out to analyse the paraspeckles formation. The control and experimental group cells were fixed and blocked in 3% BSA. Then primary antibody were added and incubated overnight at 4°C. The primary antibodies are as follows: H3K9me3 (ThemoFisher; 49−1008, 1:1000), PSPC1 (Santa Cruz; sc‐374181, 1:500), NONO (Abclonal; A5282, 1:500). The secondary corresponding fluorescence‐labelled antibody are as follow: CoraLite488‐conjugated Goat Anti‐Rabbit IgG(H+L) (Proteintech; SA00013‐2, 1:800) and CoraLite647‐conjugated AffiniPure F(ab')2 Fragment Goat Anti‐Rabbit/Mouse IgG (H+L) (Proteintech; SA00014‐9/10, 1:800). Pictures were taken by a laser scanning confocal microscope (ZEISS LSM900 and NIKON A1 HD25).

### 4.4 RNA fluorescence in situ hybridisation

RNA FISH was performed to detect NEAT1 in cells, as our previous publication.^38^ The antisense RNA probe (Table S1) is labelled by Nucleic Acid Labeling Kits (Life Technologies) with Alexa Fluor 488. Hochest was used to indicate the nucleus. Pictures were taken by a laser scanning confocal microscope (NIKON A1 HD25).

### 4.5 RNA pull‐down

RNA pull‐down with biotin‐labelled DNA probes was performed as our former publication described.^38^ Briefly, cells were collected and washed with PBS. Lysis buffer was used to resuspend the cells on ice. 20 min later, cell lysates were incubated with biotinylated sense or antisense DNA oligomers (1 μM; Table S1) corresponding to NEAT1 for 2 h, and then with 20‐μL streptavidin coupled agarose beads for 1 h. After extensive washing, the precipitated complexes were subjected to qPCR and WB.

### 4.6 Luciferase reporter assay

To detect the effect of paraspeckle formation on KIF11 promoter region, 2 kb upstream region of KIF11 gene was constructed into pGL3 reporter plasmid. pGL3‐KIF11_promoter and renilla luciferase reporter plasmid were co‐transfected into HepG2 cells. After transfection for 6 h, the above cells were treated as follows: DMSO (1:1000) for 24 h, serum starvation for 24 h, H_2_O_2_ (100 μM) for 24 h, DOXO (100 nM) for 24 h. In addition, for the detection of sh‐ctrl, sh‐NONO and sh‐PSPC1 cells, only transient co‐transfer of pGL3‐KIF11_promoter and relina plasmids is required before detection. Then a Dual‐Luciferase Reporter Assay System (Promega) was used to measure firefly and renilla luciferase activity. The detection is conducted directly 24 h after transfection. Data are represented as mean ± SD of three independent experiments.

### 4.7 ChIP assay

HepG2 cells, cultured in normal or serum‐starvation conditions, were cross‐linked with 1% formaldehyde for 10 min. The ChIP Assay Kit (Beyotime; P2083S) was used for ChIP assay. Antibodies used in ChIP assay are: Histone H3, H3K9me3 and H3K9me2 (Cell Signaling Technology; 4499S, 13969S, 4658, 1:5000). Anti‐rabbit immunoglobulin G was also used as a negative control. The qPCR was performed to analyse the bound DNA fragments. The specific primers were listed in Table S1.

### 4.8 Proteasomal degradation and protein half‐life assay

To verify whether the protein is degraded by the ubiquitin–proteasome pathway, cells were treated with or without MG132 (20 μM) for 6 h. WB was carried out to analyse the cell lysates with the indicated antibodies. To analyse the protein NONO or KIF11 half‐life, cells were treated with CHX (50 lg/mL) for the indicated periods of time. Then WB was performed to examine the NONO or KIF11 protein half‐life. Image J was used to analyse the band intensity.

### 4.9 mRNA half‐life measurement

The control (sh‐ctrl) or KIF11 knockdown (sh‐KIF11) HepG2 cells were cultured with actinomycin D (1 mg/mL) for the indicated periods, and total RNA was extracted. The qPCR was performed to examine the mRNA half‐life of indicated mRNAs.

### 4.10 Multi‐omics analysis

Shanghai OE Biotech Co., Ltd processed the mRNA sequencing and preliminary transcriptomic analysis of control or KIF11‐knockdown HepG2 cell lines. Briefly, the libraries were subjected to the Illumina HiSeq X Ten platform to generate 150 bp paired‐end reads. Raw data were first processed using Trimmomatic^117^ and the low‐quality reads were removed to obtain the clean reads, which were then retained for subsequent analyses. The sequencing data were deposited in the National Center for Biotechnology Information Gene Expression Omnibus (GEO) database. Furtherly, we used Omicshare online tools to perform GSEA and obtain relevant graphs. Tandem Mass Tags (TMT) based proteomic analysis was performed with the help of Shanghai Applied Protein Technology Company. GENEWIZ performed WGBS of ctrl and sh‐H3F3A/B HepG2 cells. Briefly, raw data were pre‐processed with fastp to obtain clean data, followed by whole‐genomic mapping and comparing via Bismark software. The MethylKit software was used to analyse and complete the detection of methylation sites in each sample. The sequencing data were deposited in the GEO database. Based on these data, we performed DMP analysis, GO analysis, KEGG analysis and visualisation analysis through Omicshare online tools and Integrative Genomics Viewer. The index numbers of sequencing data deposited in GEO database are GSE238164 and GSE239346.

### 4.11 Tumour spheres culture assay

Hepatoma cells were counted at a concentration of 1 × 10^4^ cells per well and then cultured in a stem cell culture medium (STEMCELL Technologies). A fresh medium (200 μL) was added every 2−3 days. After 10 days, 1st tumour‐spheres formed and were photographed and counted. The first generated tumour‐spheres were then digested, and the cells were counted at a concentration of 1 × 10^4^ cells per well for the second regeneration experiment. After ten days, the 2nd generated tumour‐spheres formed and were photographed and counted. The second generated tumour‐spheres were then digested, and the cells were counted at a concentration of 1 × 10^4^ cells per well for the third regeneration experiment. The 2nd and 3rd regeneration experiments were performed to detect the self‐renewal ability of hepatoma cells.

### 4.12 Gene knockout mice

*Neat1^−/−^* mice on a C57BL/6 background were generated by Biocytogen Biological Technology Co., Ltd as our previous publication.^38^ *Kif11^fl/fl^* and *Alb‐Cre^+/+^* mice on a C57BL/6 background were purchased from GemPharmatech Co. According to the existing MGI data, deletion of Kif11 results in early embryonic lethality of homozygotes, with developmental growth arrest at E3.5.^118^ In the breeding process, we did not get the homozygous mouse with liver‐specific knockout of *Kif11*, which may lead to embryo death of the mouse due to Kif11 knockout. Littermates of heterozygous mice with liver‐specific knockout of *Kif11* (*Kif11^fl/wt^;AlbCre^+/−^*) were used as our experimental mice, while littermates of heterozygous mice with *Kif11* conditional knockout (*Kif11^fl/wt^*) were used as experimental control mice. The experimental and control group mice are male (HCC occurs mainly in males, so male mice were selected as experimental mice), 6−8 weeks old. All mice were examined and ensured healthy before the initiation of the studies. Studies were conducted with approval from the Animal Research Ethics Committee of the University of Science and Technology of China (2021‐N(A)−187).

### 4.13 Hydrodynamic tail‐vein injection

We used 6 weeks old, C57BL/6, male mice for the hydrodynamic tail‐vein injection model. We injected plasmid mix encoding pT3‐c‐MYC (20 μg per mouse), pX330‐sg‐p53‐cas9 (20 μg per mouse) and pT2‐SB13 transposase (5 μg per mouse) in 2 mL saline into the tail vein of experimental and control group mice to generate liver tumours according to the previous study.^36^, ^37^ Four weeks later, mice were euthanised, and the incidence of liver cancer was calculated. Tumour weights were compared between experimental and control groups. Liver tumour tissues were used for haematoxylin–eosin and Ki67 staining.

### 4.14 In situ hybridisation and IHC analysis for clinical HCC specimens

Tissue chips containing HCC tissues, together with patient diagnosis information (Table S2) were obtained from Shanghai Outdo Biotech Company, China. HCC tissues were stained by in situ hybridisation to detect the expression level of NEAT1 and scored according to staining intensity. HCC tissues were stained by IHC to detect the expression levels of KIF11 (Proteintech; 23333‐1‐AP, 1:200), p16 (Cell Signaling Technology; 80772S, 1:200), p14 (Cell Signaling Technology; 74560S, 1:200) and scored according to staining intensity.

### 4.15 Statistics and reproducibility

All the data were repeated at least three times. Statistical analysis was carried out using GraphPad Prism to assess the differences between experimental groups. Data were analysed by Student's *t*‐test or one‐way ANOVA test followed by Tukey's multiple comparison test. *p* Values lower than .05 were statistically significant. *p < .05, **p < .01, ***p < .001.

## CONFLICT OF INTEREST STATEMENT

The authors declare no competing interests.

## FUNDING INFORMATION

This work was supported by the Fundamental Research Funds for the Central Universities (Grant No. WK9110000182, Grant No. WK9110000153), the Projects from the National Natural Science Foundation of China (Grant No. 81972307, No. U19A2008, No. 32000526, No. 82103219), the Project from the National Key R&D Program of China (Grant No. 2019YFA0709300), the Projects from China Postdoctoral Science Foundation (Grant No. 2020M682023, Grant No. 2022T150623), the Project from the Provincial Natural Science Foundation of Anhui (Grant No. 2108085QH343), the University Synergy Innovation Program of Anhui Province (Grant No. GXXT‐2019‐042) and the Science and Technology Development Program of Hangzhou (Grant No. 202204B04).

## Supporting information

Supporting Information

Supporting Information

Supporting Information

Supporting Information

Supporting Information

Supporting Information

Supporting Information

Supporting Information

Supporting Information

Supporting Information

Supporting Information

Supporting Information

## Data Availability

All data generated or analysed during this study are included in this published article and its Additional Files.
