# Supplementary figures and images for: LncRNA NEAT1 suppresses cellular senescence in hepatocellular carcinoma via KIF11‐dependent repression of CDKN2A
Source: Clin Transl Med. 2023 Sep 26;13(9):e1418. doi: 10.1002/ctm2.1418 (PMC10522973; doi:10.1002/ctm2.1418)

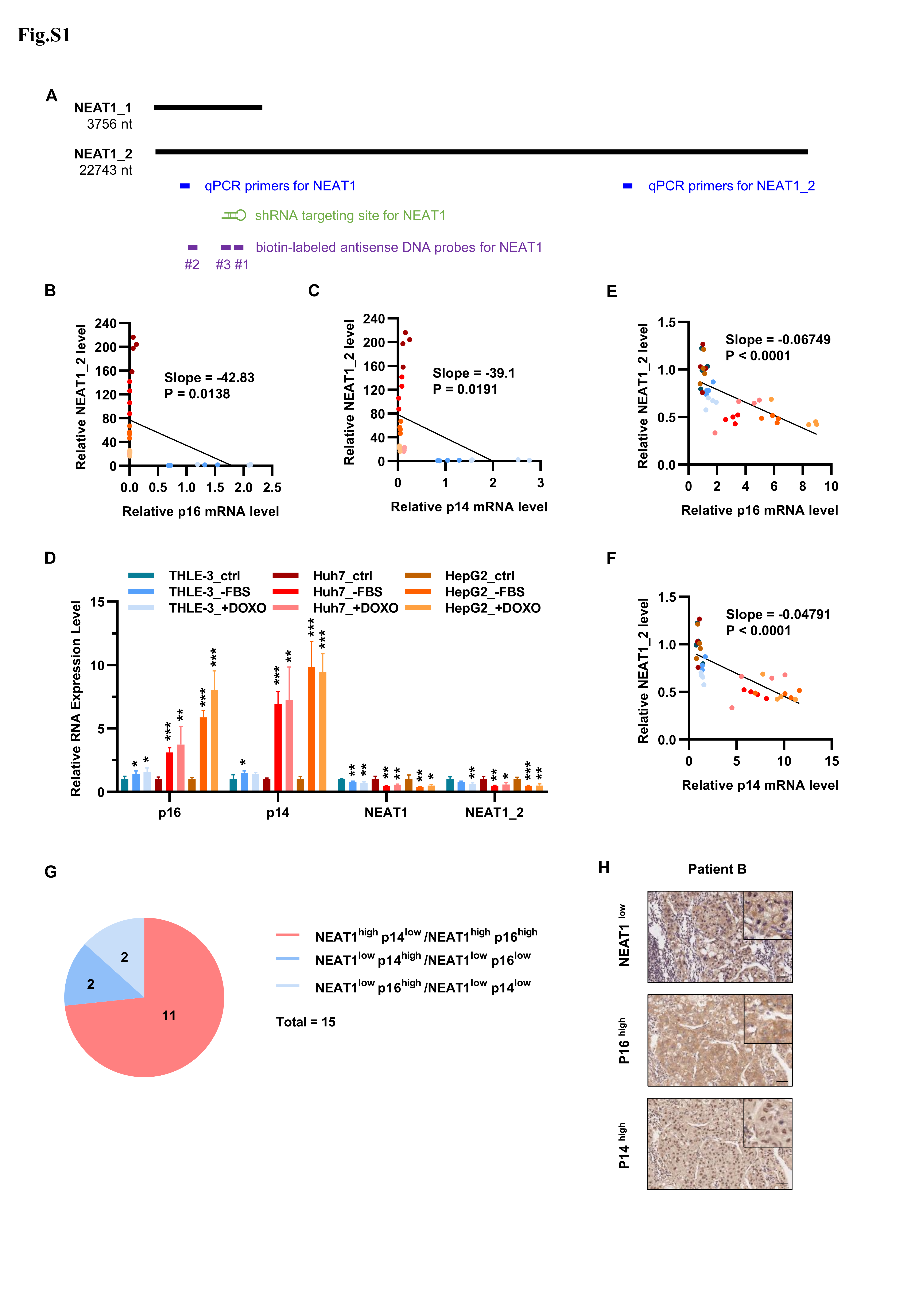

Supplement: Supplementary file 1 — Supporting Information [file CTM2-13-e1418-s002.tif]

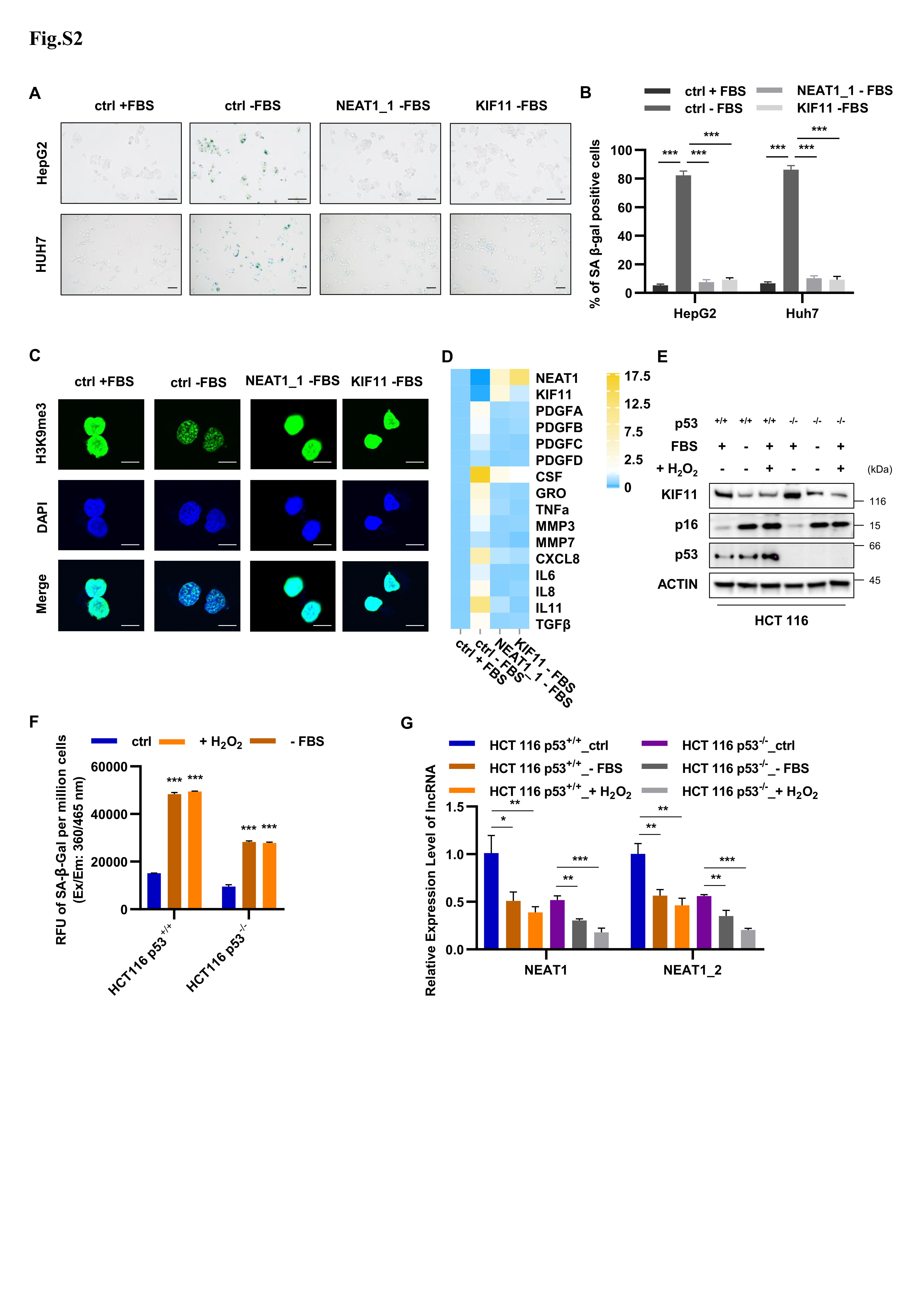

Supplement: Supplementary file 2 — Supporting Information [file CTM2-13-e1418-s005.tif]

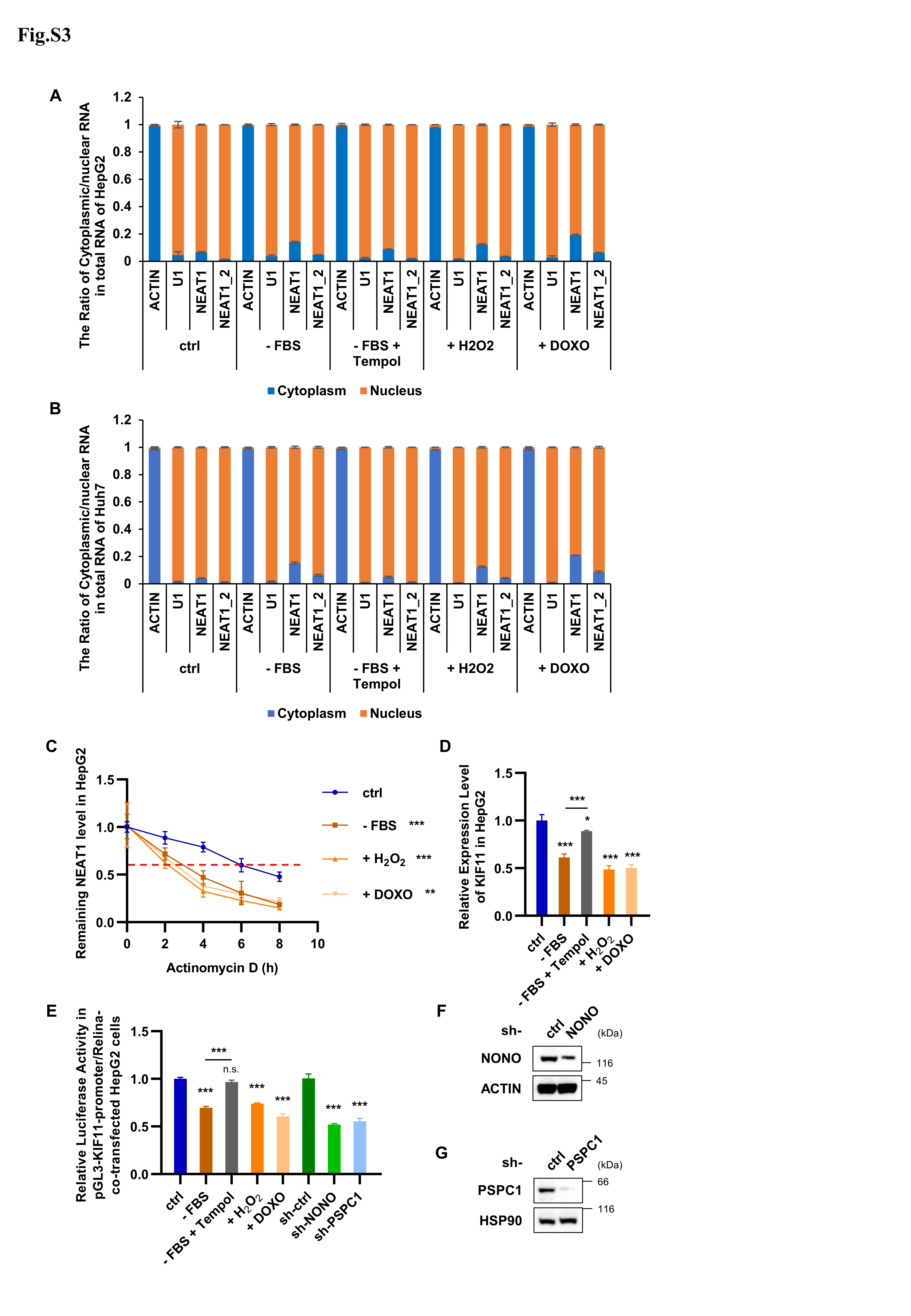

Supplement: Supplementary file 3 — Supporting Information [file CTM2-13-e1418-s009.tif]

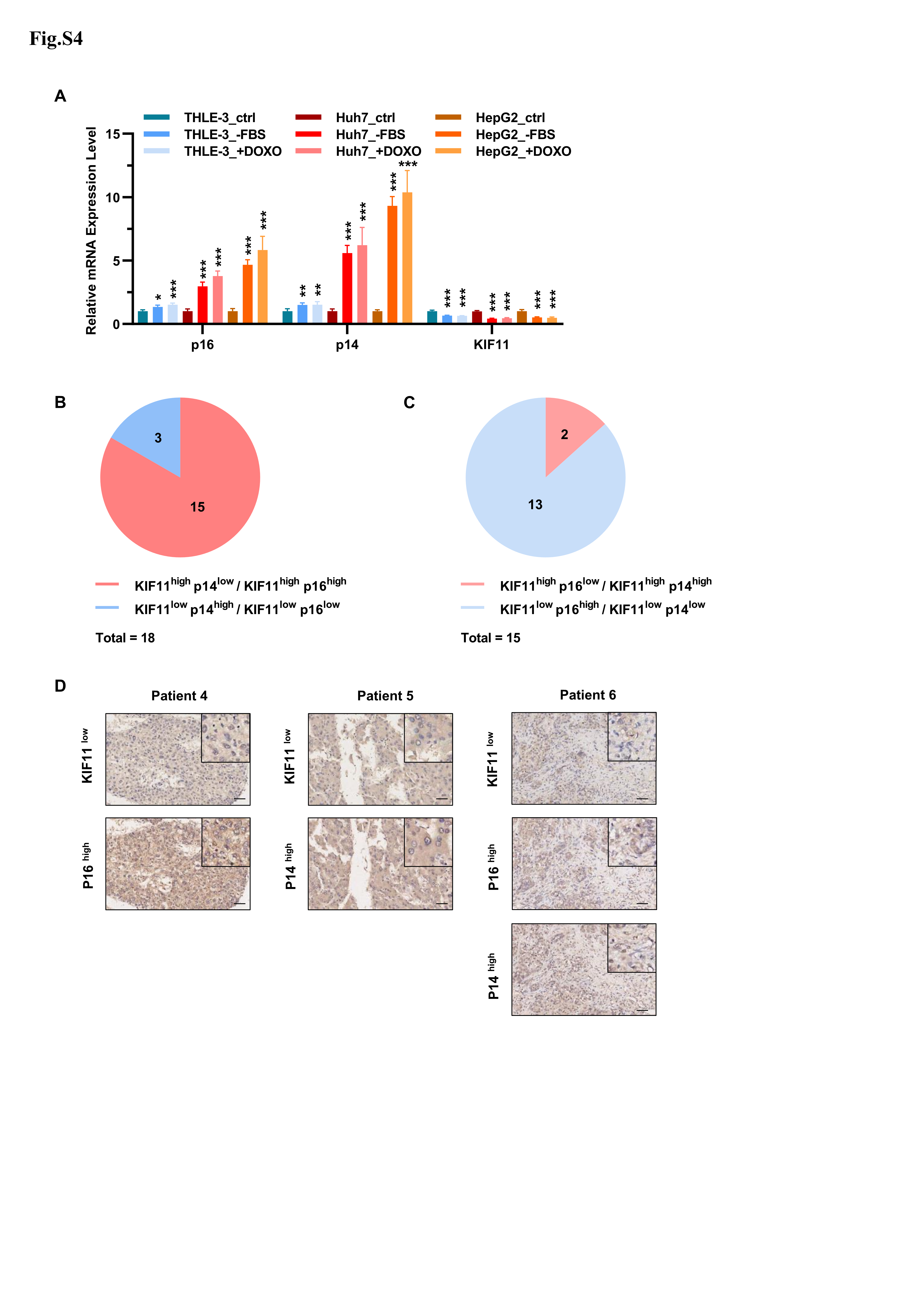

Supplement: Supplementary file 4 — Supporting Information [file CTM2-13-e1418-s006.tif]

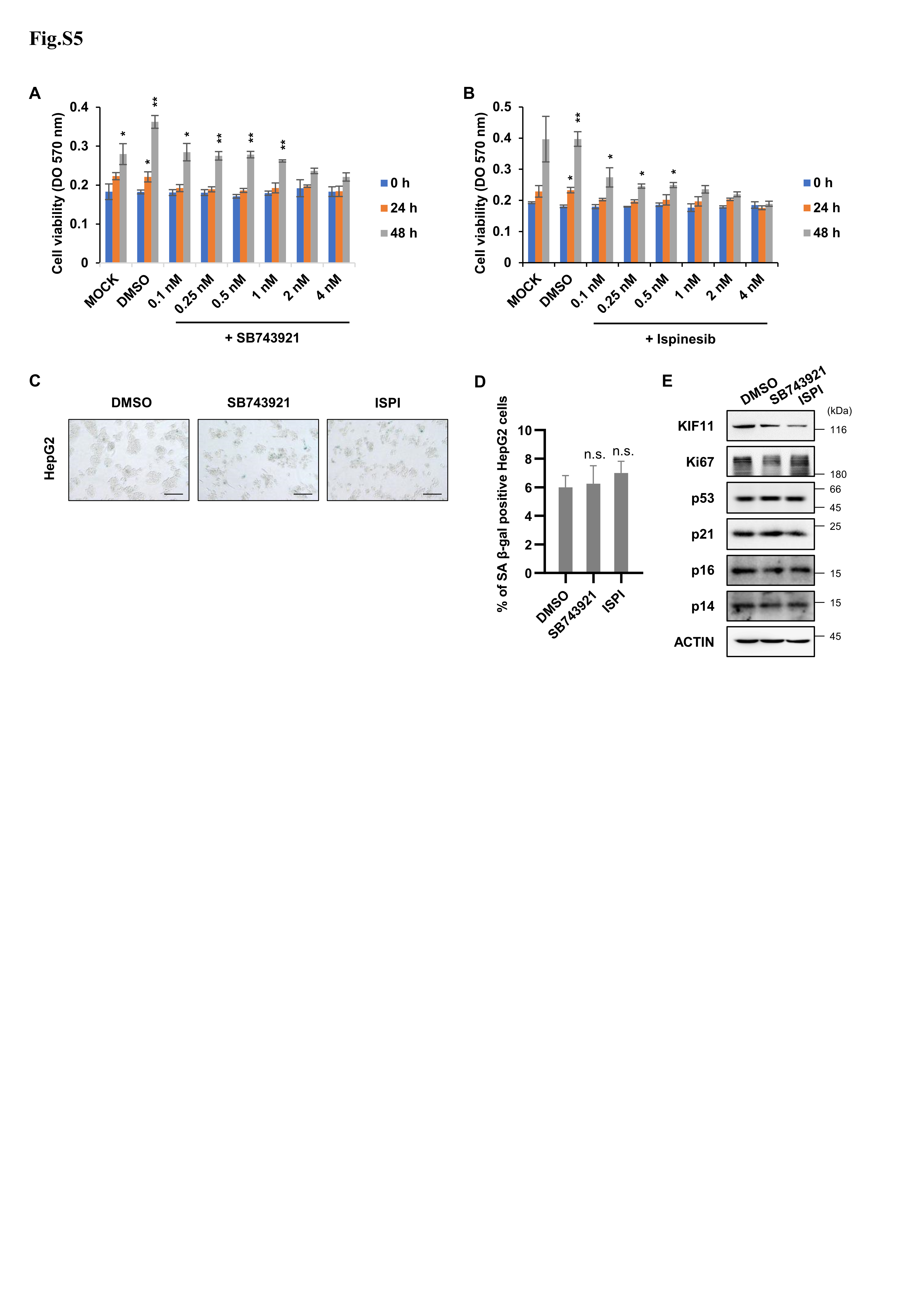

Supplement: Supplementary file 5 — Supporting Information [file CTM2-13-e1418-s012.tif]

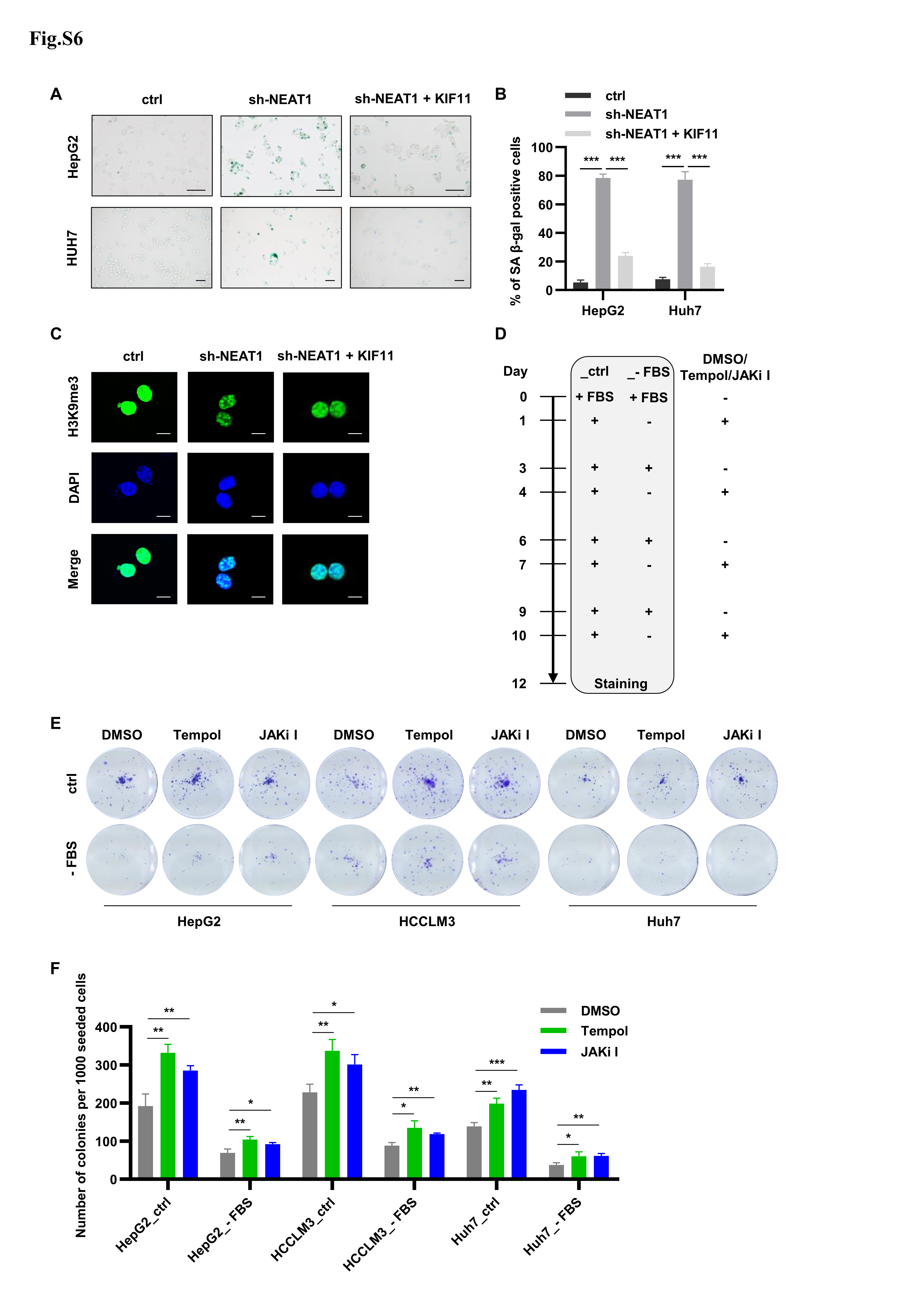

Supplement: Supplementary file 6 — Supporting Information [file CTM2-13-e1418-s007.tif]

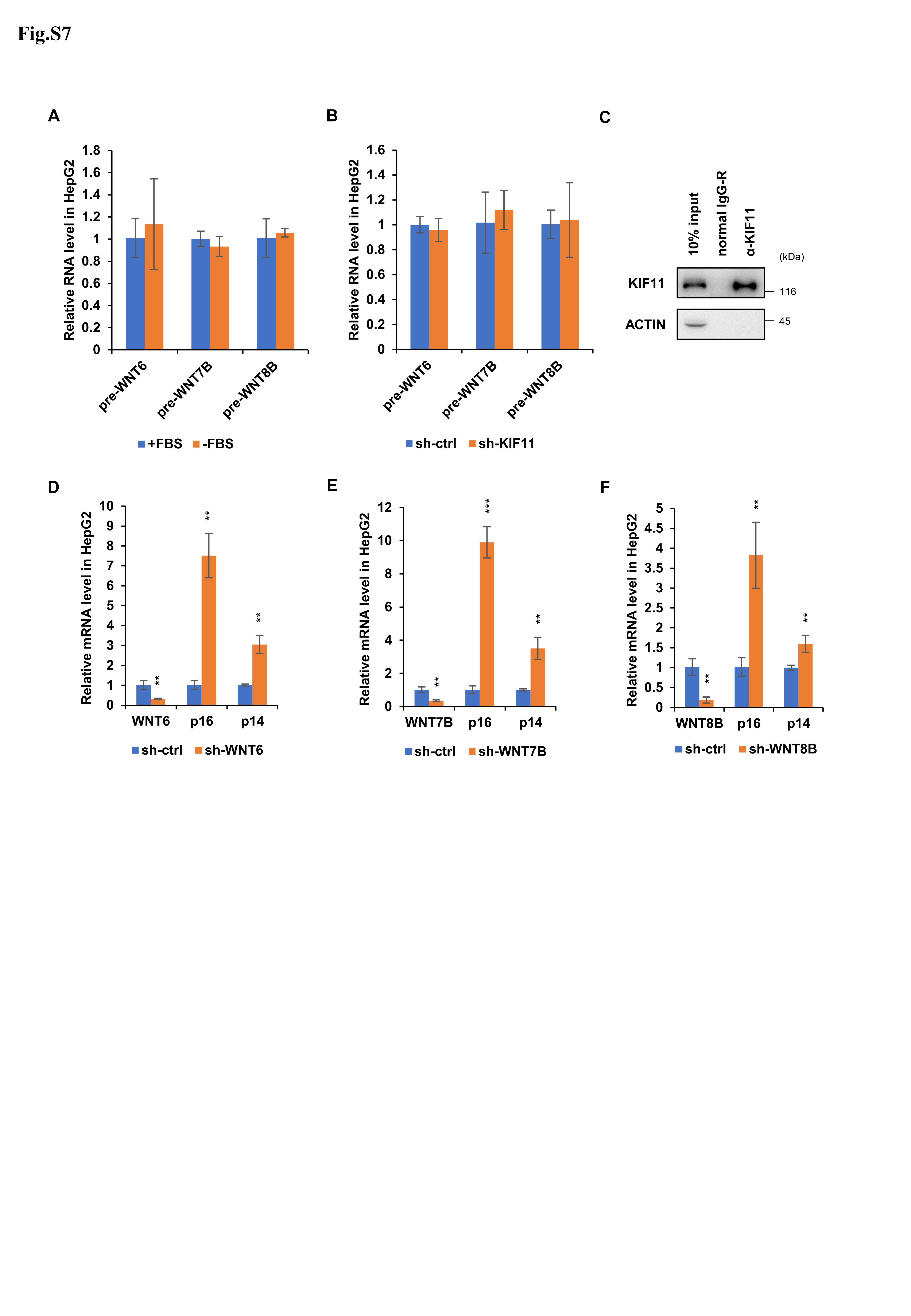

Supplement: Supplementary file 7 — Supporting Information [file CTM2-13-e1418-s008.tif]

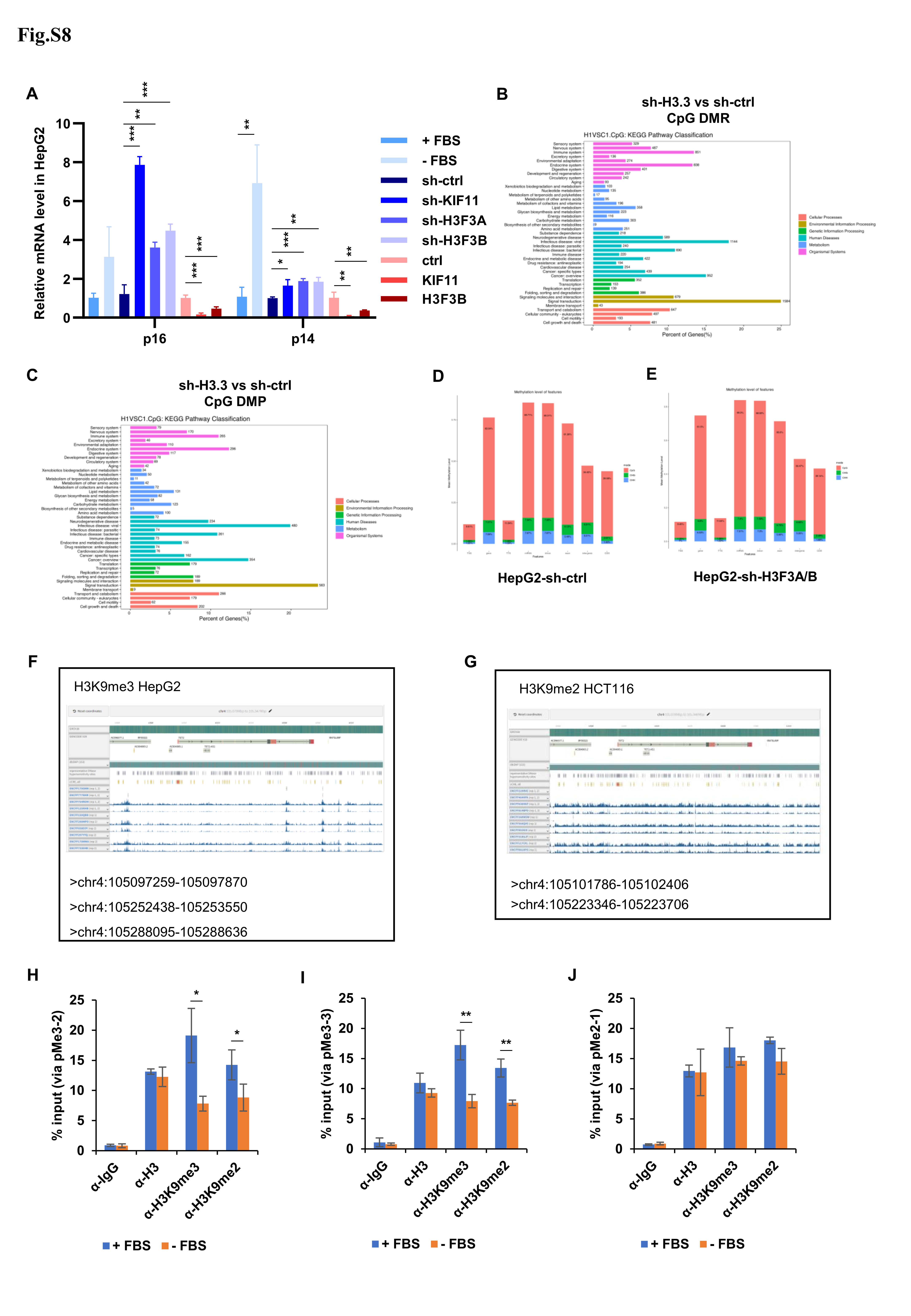

Supplement: Supplementary file 8 — Supporting Information [file CTM2-13-e1418-s001.tif]

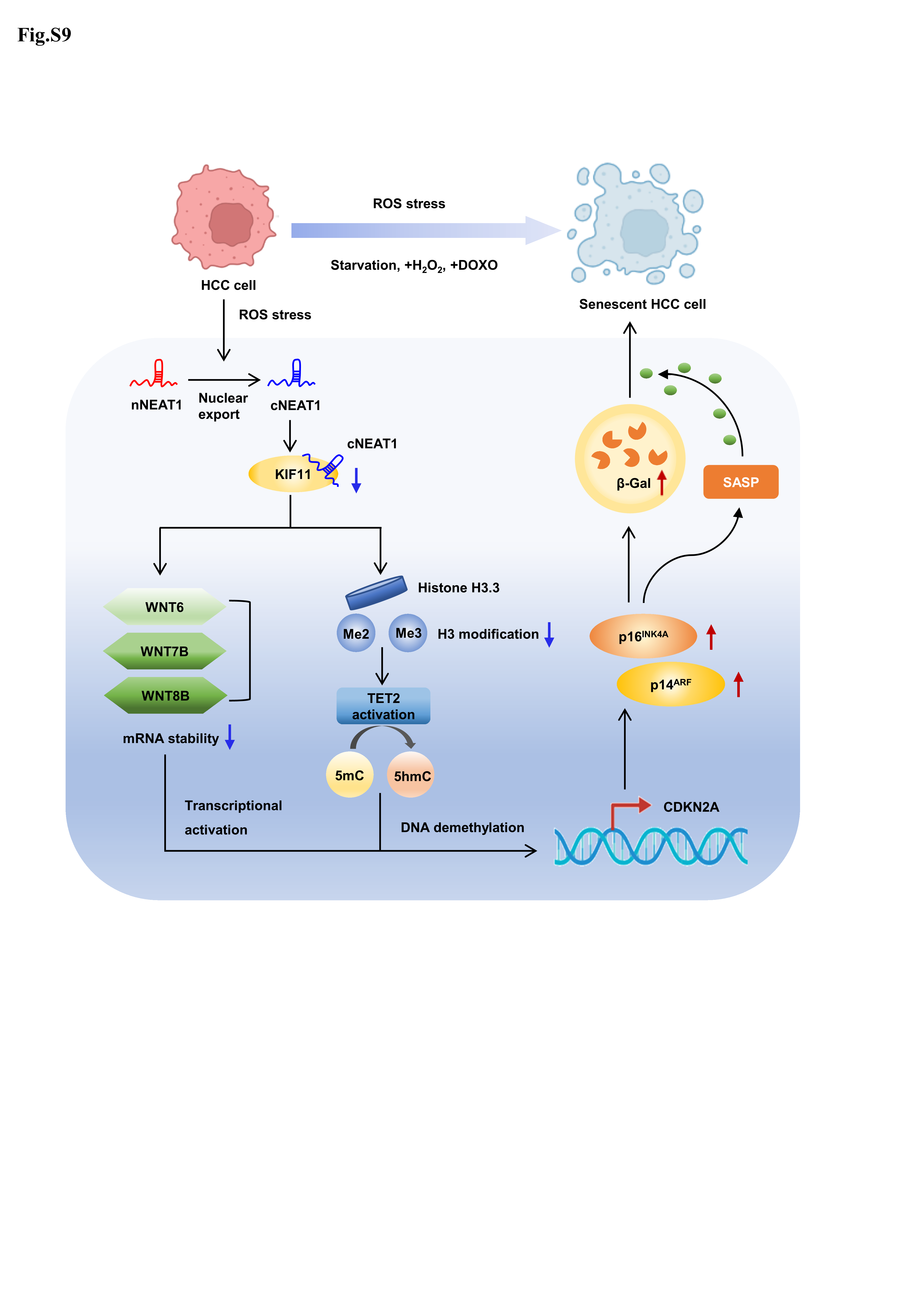

Supplement: Supplementary file 9 — Supporting Information [file CTM2-13-e1418-s010.tif]
