# Supplementary material for: LncRNA NEAT1 suppresses cellular senescence in hepatocellular carcinoma via KIF11‐dependent repression of CDKN2A
Source: Clin Transl Med. 2023 Sep 26;13(9):e1418. doi: 10.1002/ctm2.1418 (PMC10522973; doi:10.1002/ctm2.1418)
Supplement: Supplementary file 10 — Supporting Information [file CTM2-13-e1418-s004.docx]

**Supplementary Figure Legends**

**Figure S1. Supplementary figure related to Figure 1 and Figure 2.** (A) Schematic diagram of isoforms, qPCR primers, shRNA targeting site, and biotin-labeled antisense DNA probes for NEAT1 and NEAT1_2. (B) Correlation analysis of NEAT1_2 and p16 in cultured normal liver cells (HLSEC and THLE-3) and hepatoma cells (HCCLM3, Huh7, HepG2, SNU398). (C) Correlation analysis of NEAT1_2 and p14 in cultured normal liver cells (HLSEC and THLE-3) and hepatoma cells (HCCLM3, Huh7, HepG2, SNU398). (D) Expression level of NEAT1, p16 and p14 in ROS stress-induced normal liver cells (THLE-3) and hepatoma cells (Huh7 and HepG2). (E) Correlation analysis of NEAT1_2 and p16 in ROS stress-induced normal liver cells (THLE-3) and hepatoma cells (Huh7 and HepG2). (F) Correlation analysis of NEAT1_2 and p14 in ROS stress-induced normal liver cells (THLE-3) and hepatoma cells (Huh7 and HepG2). (G) Pie chart showed that 2 cases were NEAT1^high^ p14^low^/ NEAT1^high^ p16^high^, 2 cases were NEAT1^low^ p14^high^/ NEAT1^low^ p16^low^, and 11 cases were NEAT1^low^ p16^high^/ NEAT1^low^ p14^low^. A total of 15 clinical cases were analyzed. (H) IHC pictures of NEAT1^low^ p16^high^ p14^high^ expression in patient B. The scale bar indicates 50 μm.

Data shown are the mean ± SD (n ≥ 3; *p<0.05, **p<0.01, ***p<0.001, two-tailed t-test).

**Figure S2. Supplementary figure related to Figure 3 and Figure 7.** SA-β-Gal staining (A, B) and heterochromatin foci formation (C) were detected in ctrl +FBS, ctrl -FBS, NEAT1_1 -FBS, KIF11 -FBS hepatoma cells. In A, the scale bar indicates 100 μm. In C, the scale bar indicates 10 μm. (D) SASP were detected in ctrl +FBS, ctrl -FBS, NEAT1_1 -FBS, KIF11 -FBS hepatoma cells by qPCR. The average of relative expression levels were shown in the heatmap (n = 3). (E-G) The control and stress-induced HCT 116 p53^+/+^ or HCT 116 p53^-/-^ cells were analyzed by WB (E), SA-β-Gal activity assay (F), and qPCR (G).

Data shown are the mean ± SD (n ≥ 3; *p<0.05, **p<0.01, ***p<0.001, two-tailed t-test).

**Figure S3. Supplementary figure related to Figure 4 and Figure 5.** (A) HepG2 cells, treated with various conditions as shown in the figure, were analyzed for the cytoplasmic and nuclear levels of NEAT1 and NEAT1_2. (B) Huh7 cells, treated with various conditions as shown in the figure, were analyzed for the cytoplasmic and nuclear levels of NEAT1 and NEAT1_2. (C) Control and ROS stress-induced HepG2 cells were treated with actinomycin D (2.5 μM) for the indicated periods of time. Total RNAs were extracted and then analyzed by qPCR to examine the half-life of NEAT1. (D) Indicated cells were analyzed by qPCR to detect KIF11 mRNA level. (E) Indicated cells were transfected with the indicated reporter plasmid within KIF11 promoter region and Renilla luciferase plasmid. 24 hours after transfection, reporter activity was measured and plotted after normalizing with respect to Renilla luciferase activity. (F, G) HepG2 cells were transfected with lentiviruses to inhibit the expressional levels of NONO and PSPC1. The deficient efficiency was analyzed by western blotting.

Data shown are the mean ± SD (n ≥ 3; *p<0.05, **p<0.01, ***p<0.001, two-tailed t-test).

**Figure S4. Supplementary figure related to Figure 6.** (A) Expression level of KIF11, p16 and p14 in ROS stress-induced normal liver cells (THLE-3) and hepatoma cells (Huh7 and HepG2). (B) Pie chart showed that 3 cases were KIF11^high^ p14^low^/ KIF11^high^ p16^high^, and 15 cases were KIF11^low^ p14^high^/ KIF11^low^ p16^low^. A total of 18 clinical cases were analyzed. (C) Pie chart showed that 2 cases were KIF11^high^ p16^low^/ KIF11^high^ p14^high^, and 13 cases were KIF11^low^ p16^high^/ KIF11^low^ p14^low^. A total of 15 clinical cases were analyzed. (D) IHC pictures of KIF11^low^ p16^high^ expression in patient 4, KIF11^low^ p14^high^ expression in patient 5, and KIF11^low^ p16^high^ p14^high^ expression in patient 6. The scale bar indicates 50 μm.

Data shown are the mean ± SD (n ≥ 3; *p<0.05, **p<0.01, ***p<0.001, two-tailed t-test).

**Figure S5. Supplementary figure related to Figure 7.** (A, B) The classical KIF11 inhibitors (SB743921 and Ispinesib) were used to treat HepG2 cells, cell viability was detected. (C, D) SA β-galactosidase staining of DMSO, SB743921 and ISPI treated HepG2 cells. The scale bar indicates 100 μm. (E) Western Blotting were furtherly performed to detect cellular senescence-related markers.

Data shown are the mean ± SD (n ≥ 3; *p<0.05, **p<0.01, ***p<0.001, two-tailed t-test).

**Figure S6. Supplementary figure related to Figure 7.** (A-C) SA-β-Gal staining (A, B) and heterochromatin foci formation (C) were detected in ctrl, sh-NEAT1, and sh-NEAT1 + KIF11 hepatoma cells. In A, the scale bar indicates 100 μm. In C, the scale bar indicates 10 μm. (D) Illustration of the treatment and analysis procedure in the colony formation models using HepG2, HCCLM3 and Huh7 cells. (E-F) Colony formation assay was used to detect the clone formation ability of indicated cells. The data were presented in column graph.

Data shown are the mean ± SD (n ≥ 3; *p<0.05, **p<0.01, ***p<0.001, two-tailed t-test).

**Figure S7.** **Supplementary figure related to Figure 8.** (A) HepG2 cells were cultured in a serum-free medium for 0 or 48 hours. Pre-mRNA levels of WNTs were analyzed by qPCR. (B) Control and KIF11 knockdown HepG2 cells were used for qPCR to analyze the pre-mRNA levels of WNT6/7B/8B. (C) Cell lysates of HepG2 cells were incubated with normal rabbit IgG or KIF11 antibody for RIP. The immunoprecipitates were analyzed by Western blotting for protein levels. (D-F) HepG2 cells were infected with lentiviruses expressing sh-ctrl, sh-WNT6, sh-WNT7B, or sh-WNT8B. The total RNA was isolated and subjected to qPCR analysis.

Data shown are the mean ± SD (n ≥ 3; *p<0.05, **p<0.01, ***p<0.001, two-tailed t-test).

**Figure S8. Supplementary figure related to Figure 10.** (A) mRNA level of p16 and p14 in +FBS, -FBS, sh-ctrl, sh-KIF11, sh-H3F3A, sh-H3F3B, ctrl, KIF11, H3F3B HepG2 cells. WGBS analysis was performed in control and H3.3 knockdown HepG2 cells and shown in the IGV column graph. KEGG pathway analysis in genes within DMRs (B) or DMPs (C). The control (D) and H3.3 knockdown (E) HepG2 cells were subjected to genome-whole methylation sequencing. The probability and level of CpG, CHG or CHH methylation in regions such as TSS, gene, TTS, mRNA, intron, exon, intergenic and CDS in the whole genome of each cell were analyzed. (F, G) The illustrated figure of DNA modification sites around TET2 genes in the ENCODE database. (H-J) Control or serum-starved HepG2 cells were used for ChIP analysis via indicated DNA modification antibodies. And the enriched genomic DNA segments were analyzed by qPCR.

Data shown are the mean ± SD (n ≥ 3; *p<0.05, **p<0.01, ***p<0.001, two-tailed t-test).

**Figure S9. Signaling pathways involved in the process of ROS stress-induced cellular senescence in HCC.** ROS stress led to depolymerization of paraspeckle, release NEAT1 from nucleus into the cytoplasm and downregulates the transcription activity in the KIF11 promoter region. Moreover, NEAT1 competitively binds to KIF11 in the cytoplasm in senescent hepatoma cells, thus KIF11 degraded. On the one hand, KIF11 downregulation drives cellular senescence through transcriptional activation of CDKN2A by destroying the mRNA stability of WNT6/7B/8B. On the other hand, KIF11-H3.3 downregulation leads to demethylation of CDKN2A through TET2 to induce cellular senescence in HCC. These two pathways activated CDKN2A, thus induced cellular senescence in hepatoma cells.
