# Supplementary material for: LncRNA NEAT1 suppresses cellular senescence in hepatocellular carcinoma via KIF11‐dependent repression of CDKN2A
Source: Clin Transl Med. 2023 Sep 26;13(9):e1418. doi: 10.1002/ctm2.1418 (PMC10522973; doi:10.1002/ctm2.1418)
Supplement: Supplementary file 11 — Supporting Information [file CTM2-13-e1418-s003.docx]

**Supplementary Methods**

Cell culture

HepG2, Huh7, HCCLM3, PLC, and HEK293T cell lines were cultured in Dulbecco’s modified Eagle’s medium (DMEM) complete medium. SNU398 cell lines were cultured in Roswell Park Memorial Institute (RPMI) 1640 complete medium. THLE-3 cell lines were cultured in Bronchial Epithelial Cell Growth Medium (BEGM), discarding the GA and Epinephrine, and adding EGF5 (ng/mL), phosphoethanolamine (70 ng/mL), and FBS. Human sinusoidal endothelial cells (HSEC), purchased from Zhejiang Meisen Cell Technology Co., Ltd, were cultured in Endothelial Cell Medium (ScienCell, 1001). All cells were tested by Cell Culture Contamination Detection Kit (ThermoFisher) to ensure that cells have no mycoplasma contamination.

Generation of the lentiviral particles

The human H3F3A and H3F3B sequences were cloned into the lentivirus expression vector pCDH-CMV-MCS-EF1-Puro. The human KIF11 sequence was cloned into the lentivirus expression vector pSIN-3×FLAG. The DNA fragments containing various shRNAs were cloned into the lentivirus knockdown vector pLKO.1. The DNA oligos and primers are listed in Supplementary Table S1. The lentivirus functional vector and the packaging plasmids (psPAX2 and pMD2.G) were co-transfected into HEK293T cells, using Lipofectamine 2000 (Invitrogen). 48 hours later, the virus particles were collected. Then, recombinant lentivirus transducing units were used to infect the indicated cells using 1 μg/ml polybrene (Sigma-Aldrich). 48 hours after infection, puromycin (25 μg/mL) was added to selected positive cells.

Colony formation assay

Indicated cells (2 × 10^3^) were cultured in twelve-well plates. Fourteen days later, cells were fixed with 4% paraformaldehyde, and then stained with crystal violet for 1h, following by washing with PBS. The cell colonies were photographed, counted and shown in column graph as the mean ± SD.

Real-time qPCR

SYBR Green real-time PCR analysis kit (Takara) was used for qPCR. Specific primers were listed in Supplementary Table S1. The recorded cycle threshold (Ct) was normalized against an internal control (β-action).

Western blot

Cells were cultured and treated as indicated. Total protein was extracted with RIPA lysis buffer (Beyotime), and normalized using Lowry Protein Assay Kit (Solar). Primary antibodies: β-actin (Proteintech, 81115-1-RR, 1:10000), H3.3 (Proteintech, 13754-1-AP, 1:1000), KIF11 for human (Proteintech, 23333-1-AP, 1:1000) , WNT6 (Proteintech, 24201-1-AP, 1:1000), HSP90 (Proteintech, 13171-1-AP, 1:2000), Ki67 (Proteintech, 27309-1-AP, 1:2000), WNT7B (Abclonal, A17004, 1:1000), NONO (Abclonal, A5282, 1:1000), WNT8B (R&D System, AF3367, 1:1000), Histone H3 (Cell Signaling Technology, 4499S, 1:2000), Histone H3.1/H3.2 (ACTIVE MOTIF, 61629, 1:1000), H3K9me3 (Cell Signaling Technology, 13969S, 1:1000), H3K9me2 (Cell Signaling Technology, 4658, 1:1000), p16 INK4A (Cell Signaling Technology, 80772S, 1:1000); p21 (Abcam, ab188224, 1:1000), p27 KIP 1 (Abcam, ab32034, 1:1000); PSPC1 (Santa Cruz, sc-374387, 1:200), SFPQ (Santa Cruz, sc-101137, 1:200), mouse-specific Kif11 (Santa Cruz, sc-365593, 1:200), p53 (Santa Cruz, sc-126, 1:200), p14 ARF (Cell Signaling Technology, 74560S, 1:1000), TET1 (Santa Cruz, sc-293186, 1:200), TET2 (Proteintech, 21207-1-AP, 1:1000), TET3 (Santa Cruz, sc-518126, 1:200), Lamin B1 (Proteintech, 12987-1-AP, 1:1000).

Dot blot assay

Genomic DNA was extracted from indicated cell lines and then was dropped onto the nitrocellulose membrane for dot blot assay (40 ng/2 μL). After dried at room temperature, the membrane was blocked in 5% BSA with TBST for 1 h, and incubated in TBST with 5mC antibody (Cell Signaling Technology, 28692S, 1:1000) or 5hmC antibody (Cell Signaling Technology, 51660S, 1:1000) for another 6 hours, at 4 ℃. After washed 3 times with TBST, the membrane was incubated with the secondary antibody (Peroxidase AffiniPure Goat Anti-Rabbit IgG (H+L), Jackson ImmunoResearch, 111-035-003, 1:100000) in TBST for 1 hour. After washing with TBST for another 3 times, the membrane was used for ECL analysis.

Cytosolic and nuclear fractionation

Indicated cells were incubated in a hypotonic buffer on ice for 5 min. An equal volume of hypotonic buffer containing 1% NP-40 was then added. 5 minutes later, the supernatant was then centrifugated at 5000×g for 5 min. Cytosolic fraction was collected, and the pellets were resuspended in nucleus resuspension buffer and incubated on ice for 30 min. After centrifugation at 12,000×g for 10 min, insoluble membrane debris were removed, and then nuclear fraction was collected.
