# Supplementary material for: LncRNA NEAT1 suppresses cellular senescence in hepatocellular carcinoma via KIF11‐dependent repression of CDKN2A
Source: Clin Transl Med. 2023 Sep 26;13(9):e1418. doi: 10.1002/ctm2.1418 (PMC10522973; doi:10.1002/ctm2.1418)
Supplement: Supplementary file 12 — Supporting Information [file CTM2-13-e1418-s011.docx]

**Supplementary Table S1. Oligomers used in this study.**

| **Name** | **Application** | **Sequence** |
| --- | --- | --- |
| qPCR-H3F3A-F | qPCR | AAAGCCGCTCGCAAGAGTGCG |
| qPCR-H3F3A-R | qPCR | ACTTGCCTCCTGCAAAGCAC |
| qPCR-H3F3B-F | qPCR | TGAGGTTTCAGAGCGCAGCCAT |
| qPCR-H3F3B-R | qPCR | GCGAGCCAACTGGATGTCTTTG |
| qPCR-KIF11-F | qPCR | ATCAATTGGCGGGGTTCCAT |
| qPCR-KIF11-R | qPCR | CTGGGCTCGCAGAGGTAATC |
| qPCR-p53-F | qPCR | GAGCTGAATGAGGCCTTGGA |
| qPCR-p53-R | qPCR | CTGAGTCAGGCCCTTCTGTCTT |
| qPCR-p27-F | qPCR | GGCCTCAGAAGACGTCAAAC |
| qPCR-p27-R | qPCR | ACAGGATGTCCATTCCATGA |
| qPCR-p21-F | qPCR | CCGGCGAGGCCGGGATGAG |
| qPCR-p21-R | qPCR | CTTCCTCTTGGAGAAGATC |
| qPCR-p16-F | qPCR | AGCATGGAGCCTTCGGCTGA |
| qPCR-p16-R | qPCR | CCATCATCATGACCTGGATCG |
| qPCR-p14-F | qPCR | CTGTGGCCCTCGTGCTGAT |
| qPCR-p14-R | qPCR | CGTGTCCAGGAAGCCCTC |
| qPCR-MDM2-F | qPCR | GCAGTGAATCTACAGGGACGC |
| qPCR-MDM2-R | qPCR | ATCCTGATCCAACCAATCACC |
| qPCR-CDK1-F | qPCR | TCCGCAACAGGGAAGAAC |
| qPCR-CDK1-R | qPCR | GAGCCTTTTTAGATGGCTGCT |
| qPCR-CDK2-F | qPCR | CTTTGGAGTCCCTGTCCGTA |
| qPCR-CDK2-R | qPCR | CGAAAGATCCGGAAGAGTTG |
| qPCR-CDK4-F | qPCR | TGCACAGTGTCACGAACAGA |
| qPCR-CDK4-R | qPCR | ACCTCGGAGAAGCTGAAACA |
| qPCR-CDK6-F | qPCR | CATCGTTCACCGAGATCTGA |
| qPCR-CDK6-R | qPCR | CCAACACTCCACATGTCCAC |
| qPCR-Wnt6-F | qPCR | GGCAGCCCCTTGGTTATGG |
| qPCR-Wnt6-R | qPCR | CTCAGCCTGGCACAACTCG |
| qPCR-Wnt7b-F | qPCR | CACAGAAACTTTCGCAAGTGG |
| qPCR-Wnt7b-R | qPCR | GTACTGGCACTCGTTGATGC |
| qPCR-Wnt8b-F | qPCR | CCGACACCTTTCGCTCCATC |
| qPCR-Wnt8b-R | qPCR | CAGCCCTAGCGTTTTGTTCTC |
| qPCR-preWNT6-F | qPCR | agaatctcacccctgctgtc |
| qPCR-preWNT6-R | qPCR | gggaatgggaggtgaaagga |
| qPCR-preWNT7b-F | qPCR | ggcccttcgtctctatccat |
| qPCR-preWNT7b-R | qPCR | atacgactcggagcagctac |
| qPCR-preWNT7b-F | qPCR | gagatgagggccccagatac |
| qPCR-preWNT7b-R | qPCR | gtctgtcccacacctgagaa |
| qPCR-TET1-F | qPCR | CAGAACCTAAACCACCCGTG |
| qPCR-TET1-R | qPCR | TGCTTCGTAGCGCCATTGTAA |
| qPCR-TET2-F | qPCR | ATACCCTGTATGAAGGGAAGCC |
| qPCR-TET2-R | qPCR | CTTACCCCGAAGTTACGTCTTTC |
| qPCR-TET3-F | qPCR | TCCAGCAACTCCTAGAACTGAG |
| qPCR-TET3-R | qPCR | AGGCCGCTTGAATACTGACTG |
| qPCR-CD133-F | qPCR | ACCAGGTAAGAACCCGGATCAA |
| qPCR-CD133-R | qPCR | CAAGAATTCCGCCTCCTAGCACT |
| qPCR-CD326-F | qPCR | GCCAGTGTACTTCAGTTGGTGC |
| qPCR-CD326-R | qPCR | CCCTTCAGGTTTTGCTCTTCTCC |
| qPCR-ALDH1-F | qPCR | CTGCTGGCGACAATGGAGT |
| qPCR-ALDH1-R | qPCR | GTCAGCCCAACCTGCACAG |
| qPCR-NEAT1-F | qPCR | cccttcttcctccctttaact |
| qPCR-NEAT1-R | qPCR | cctctcttcctccaccattac |
| qPCR-NEAT1_2-F | qPCR | agtagcctctggtgtcatttg |
| qPCR-NEAT1_2-R | qPCR | gacacttctccagggaacatac |
| Biotin-NEAT1_probe-sense-1 | RNA pulldown | Biotin-agccttgtaaatgcctatatt |
| Biotin-NEAT1_probe-antisense-1 | RNA pulldown | Biotin-aatataggcatttacaaggct |
| Biotin-NEAT1_probe-sense-2 | RNA pulldown | Biotin-TCATGGACCGTGGTTTGTTACTATAGTGT |
| Biotin-NEAT1_probe-antisense-2 | RNA pulldown | Biotin-ACACTATAGTAACAAACCACGGTCCATGA |
| Biotin-NEAT1_probe-sense-3 | RNA pulldown | Biotin-AAGTGAGAAGTTGCTTAGAAACTTTCC |
| Biotin-NEAT1_probe-antisense-3 | RNA pulldown | Biotin-GGAAAGTTTCTAAGCAACTTCTCACTT |
| Kif11-5wt-tF1 | Genotyping | AAGCGTGGAGTGAGCCATCTCACT |
| Kif11-5wt-tR1 | Genotyping | TGCCCAAAATTCATTTAATAGCCAAGC |
| Kif11-3wt-tF1 | Genotyping | GCTGGGAATTGCACTTAGGACCTCT |
| Kif11-3wt-tR1 | Genotyping | GGACTCCAGGGTATTTGGAATGATAG |
| H11-tF3 | Genotyping | GGGCAGTCTGGTACTTCCAAGCT |
| 000119-Alb-tR1 | Genotyping | TAGCTACCTATGCGATCCAAACAAC |
| H11-tR3 | Genotyping | ATATCCCCTTGTTCCCTTTCTGC |
| ChIP-H3k9me3-1-F | ChIP-qPCR | ACCTGTCAGCCTGCCCTACA |
| ChIP-H3k9me3-1-R | ChIP-qPCR | CTGAGCTCGTGATCCGCCTG |
| ChIP-H3k9me3-2-F | ChIP-qPCR | TGACAGTGCCATTTGCAGAACA |
| ChIP-H3k9me3-2-R | ChIP-qPCR | TGCAAAATGGGTCACAGCCCT |
| ChIP-H3k9me3-3-F | ChIP-qPCR | AGGGTGAGTGGGGATGCAGA |
| ChIP-H3k9me3-3-R | ChIP-qPCR | TGGTGCCAGCTGTTTGGGAG |
| ChIP-H3k9me2-1-F | ChIP-qPCR | TCTACTTTGTTGCACAATGTCCAGT |
| ChIP-H3k9me2-1-R | ChIP-qPCR | AAGCAGAACTCTTGTCAACTGCT |
| ChIP-H3k9me2-2-F | ChIP-qPCR | ACCTCAAGAGTGAGACTTGGCA |
| ChIP-H3k9me2-2-R | ChIP-qPCR | TGTGTGCAATGCTATGGACTTCA |
| sh-NEAT1-F | plasmid construction | ccggagccttgtaaatgcctatattctcgagaatataggcatttacaaggcttttttg |
| sh-NEAT1-R | plasmid construction | aattcaaaaaagccttgtaaatgcctatattctcgagaatataggcatttacaaggct |
| sh-NONO-F | plasmid construction | CCGGGCAGGCGAAGTCTTCATTCATCTCGAGATGAATGAAGACTTCGCCTGCTTTTTG |
| sh-NONO-R | plasmid construction | AATTCAAAAAGCAGGCGAAGTCTTCATTCATCTCGAGATGAATGAAGACTTCGCCTGC |
| sh-PSPC1-F | plasmid construction | CCGGGCCTTGACTGTCAAGAACCTTCTCGAGAAGGTTCTTGACAGTCAAGGCTTTTTTG |
| sh-PSPC1-R | plasmid construction | AATTCAAAAAAGCCTTGACTGTCAAGAACCTTCTCGAGAAGGTTCTTGACAGTCAAGGC |
| sh-KIF11-F | plasmid construction | CCGGGCGCCCATTCAATAGTAGAATCTCGAGATTCTACTATTGAATGGGCGCTTTTTG |
| sh-KIF11-R | plasmid construction | aattcaaaaaGCGCCCATTCAATAGTAGAATCTCGAGATTCTACTATTGAATGGGCGC |
| sh-H3F3A-F | plasmid construction | CCGGGAACTTCTGATTCGCAAACTTCTCGAGAAGTTTGCGAATCAGAAGTTCTTTTTG |
| sh-H3F3A-R | plasmid construction | aattcaaaaaGAACTTCTGATTCGCAAACTTCTCGAGAAGTTTGCGAATCAGAAGTTC |
| sh-H3F3B-F | plasmid construction | CCGGGCTTCGAGAGATTCGTCGTTACTCGAGTAACGACGAATCTCTCGAAGCTTTTTG |
| sh-H3F3B-R | plasmid construction | aattcaaaaaGCTTCGAGAGATTCGTCGTTACTCGAGTAACGACGAATCTCTCGAAGC |
| sh-WNT6-F | plasmid construction | CCGGGAGCGTTTAAAGGACACTGTACTCGAGTACAGTGTCCTTTAAACGCTCTTTTTG |
| sh-WNT6-R | plasmid construction | aattcaaaaaGAGCGTTTAAAGGACACTGTACTCGAGTACAGTGTCCTTTAAACGCTC |
| sh-WNT7B-F | plasmid construction | CCGGCCCGATGCCATCATTGTGATTCTCGAGAATCACAATGATGGCATCGGGTTTTTG |
| sh-WNT7B-R | plasmid construction | aattcaaaaaCCCGATGCCATCATTGTGATTCTCGAGAATCACAATGATGGCATCGGG |
| sh-WNT8B-F | plasmid construction | CCGGCCCAGAGTGGTATTGAAGAATCTCGAGATTCTTCAATACCACTCTGGGTTTTTG |
| sh-WNT8B-R | plasmid construction | aattcaaaaaCCCAGAGTGGTATTGAAGAATCTCGAGATTCTTCAATACCACTCTGGG |
| RNA probe-NEAT1 | RNA-FISH | UGGCAUGGACAAGUUGAAGAUUAGCCCUCCCGGCCCUCCUGCAGCCCUGCACCCACUGCCUGCCUUCCUGAUCAUUUCCAGGGCUGCUGCGGCCUAUUCCUCCUGACUCCUCCACCCCUUCUACCUCUCCCUGCCUUCCUCCUUCCACACAGACCAGCGCACCCGGGCUCGCUCAGCUAUGCAAGAGCGGCGCCCUCCCAGAGGUCAAGUUCCCCCUCCACCAGGCAGUAGGGACAGCCUGGCCUGGAGCGUGGCUGUUCUGCCUGGGGGACCCUGCGGAUAUUUUCCAUGCAGCCUGCCCCACUGUGGUCCCCCUAGACCUAGUCUCCUUGCCAAGCUUCCUUCUCGCACCCCCAGCCCGCCCCCUCGUCUCAUCUAACUCAGUCAUCUCUCCCUGUCUGUCCCCUGAAGCCCUGAGCUAGCCACUUCCUCCCCCACAACUA |

**Supplementary Table S2. Detailed clinical patient information of HCC tissues used in this study.**

| ***Patients*** | **Age** | **Gender** | **pTNM:T**  **(AJCC 8)** | **Stage (AJCC 8)** | **Survival State** | **Follow-up Date** | **Number** | **Size**  **(cm)** | **Recurrence** |
| --- | --- | --- | --- | --- | --- | --- | --- | --- | --- |
| *P1* | 73 | Female | T1 | 1 | Death | 2010-05 | 1 | 4 | Yes |
| *P2* | 37 | Female | T1 | 1 | Survival | 2012-02 | 1 | 1.6 | No |
| *P3* | 53 | Male | T1 | 1 | Death | 2009-01 | 3 | 4.5 | Yes |
| *P4* | 41 | Male | T2 | 2 | Survival | 2012-02 | 2 | 7 | Yes |
| *P5* | 80 | Female | T2 | 2 | Death | 2008-02 | 1 | 7 | Yes |
| *P6* | 56 | Male | T1 | 1 | Survival | 2012-02 | 1 | 1.8 | No |
| *P7* | 41 | Male | T1 | 1 | Death | 2010-09 | 1 | 3 | Yes |
| *P8* | 52 | Male | T2 | 2 | Survival | 2012-02 | 1 | 4 | Yes |
| *P9* | 49 | Male | T2 | 2 | Death | 2010-03 | 1 | 4.5 | Yes |
| *P10* | 50 | Male | T2 | 2 | Death | 2007-12 | 2 | 5.5 | No |
| *P11* | 43 | Female | T1 | 1 | Survival | 2012-02 | 1 | 3 | No |
| *P12* | 63 | Male | T1 | 1 | Death | 2011-03 | 1 | 4 | Yes |
| *P13* | 43 | Male | T1 | 1 | Survival |  | 1 | 7 | Yes |
| *P14* | 38 | Male | T1 | 1 | Death | 2008-01 | 1 | 7 | Yes |
| *P15* | 45 | Male | T1 | 1 | Death | 2007-07 | 4 | 12 | Yes |
| *P16* | 59 | Female | T2 | 2 | Death | 2009-10 | 1 | 8 | Yes |
| *P17* | 56 | Male | T1 | 1 | Survival | 2012-02 | 1 | 8 | No |
| *P18* | 46 | Male | T2 | 2 | Survival | 2012-02 | 1 | 3.5 | No |
| *P19* | 27 | Male | T2 | 2 | Death | 2009-12 | 1 | 11 | Yes |
| *P20* | 54 | Male | T1 | 1 | Death | 2008-04 | 1 | 3.5 | Yes |
| *P21* | 50 | Male | T1 | 1 | Survival | 2012-02 | 2 | 2.8 | Yes |
| *P22* | 59 | Male | T2 | 2 | Survival | 2012-02 | 1 | 12 | No |
| *P23* | 59 | Male | T2 | 2 | Death | 2008-02 | 1 | 12 | Yes |
| *P24* | 44 | Male | T1 | 1 | Survival | 2012-02 | 1 | 4 | No |
| *P25* | 39 | Male | T1 | 1 | Death | 2007-07 | 1 | 5 | Yes |
| *P26* | 70 | Female | T2 | 2 | Survival | 2012-02 | 1 | 10.5 | No |
| *P27* | 51 | Male | T1 | 1 | Death | 2009-04 | 1 | 3 | No |
| *P28* | 54 | Male | T1 | 1 | Death | 2007-10 | 1 | 9 | Yes |
| *P29* | 48 | Male | T2 | 2 | Death | 2007-04 | 1 | 9 | Yes |
| *P30* | 35 | Male | T2 | 2 | Death | 2010-10 | 3 | 2.5 | Yes |
| *P31* | 53 | Male | T1 | 1 | Death | 2007-11 | 1 | 7 | Yes |
| *P32* | 48 | Male | T1 | 1 | Death | 2008-02 | 3 | 5 | Yes |
| *P33* | 46 | Male | T1 | 1 | Survival | 2012-02 | 1 | 2.5 | Yes |
| *P34* | 29 | Male | T1 | 1 | Survival |  | 1 | 3 | No |
| *P35* | 62 | Male | T1 | 1 | Survival | 2012-02 | 2 | 5.5 | No |
| *P36* | 57 | Male | T1 | 1 | Survival | 2012-02 | 1 | 4 | No |
| *P37* | 79 | Male | T2 | 2 | Death | 2008-05 | 1 | 14 | Yes |
| *P38* | 39 | Male | T1 | 1 | Survival | 2012-02 | 2 | 5.5 | No |
| *P39* | 39 | Male | T1 | 1 | Death | 2008-12 | 2 | 6.5 | Yes |
| *P40* | 55 | Female | T1 | 1 | Survival | 2012-02 | 1 | 8 | No |
| *P41* | 59 | Male | T1 | 1 | Survival | 2012-02 | 1 | 2.5 | Yes |
| *P42* | 60 | Female | T1 | 1 | Survival | 2012-02 | 2 | 1 | Yes |
| *P43* | 59 | Male | T1 | 1 | Survival | 2012-02 | 1 | 2 | No |
| *P44* | 75 | Male | T1 | 1 | Death | 2007-11 | 1 | 3.5 | Yes |
| *P45* | 66 | Male | T2 | 2 | Death | 2010-03 | 1 | 3.5 | No |
| *P46* | 34 | Male | T1 | 1 | Survival | 2012-02 | 1 | 1.2 | No |
| *P47* | 41 | Male | T1 | 1 | Death | 2008-03 | 1 | 4.5 | Yes |
| *P48* | 57 | Male | T2 | 2 | Death | 2008-02 | 1 | 8 | No |
| *P49* | 59 | Male | T2 | 2 | Death | 2009-02 | 1 | 10 | Yes |
| *P50* | 51 | Male | T1 | 1 | Survival | 2012-02 | 1 | 3.5 | Yes |
| *P51* | 84 | Male | T2 | 2 | Death | 2008-07 | 1 | 5.5 | Yes |
| *P52* | 39 | Male | T1 | 1 | Survival | 2012-02 | 1 | 6.5 | Yes |
| *P53* | 60 | Male | T1 | 1 | Death | 2011-02 | 1 | 2.2 | Yes |
| *P54* | 40 | Male | T1 | 1 | Survival | 2012-02 | 1 | 11 | Yes |
| *P55* | 35 | Male | T1 | 1 | Death | 2008-03 | 1 | 2.5 | No |
| *P56* | 60 | Female | T1 | 1 | Survival | 2012-02 | 1 | 6.5 | No |
| *P57* | 50 | Male | T1 | 1 | Survival | 2012-02 | 1 | 3 | Yes |
| *P58* | 63 | Male | T2 | 2 | Death | 2009-08 | 1 | 14 | Yes |
| *P59* | 42 | Female | T1 | 1 | Survival | 2012-02 | 1 | 2 | No |
| *P60* | 58 | Male | T2 | 2 | Death | 2012-06 | 1 | 3 | Yes |
| *P61* | 70 | Female | T2 | 2 | Survival | 2012-02 | 1 | 4.5 | No |
| *P62* | 41 | Male | T1 | 1 | Survival | 2012-02 | 1 | 3.8 | No |
| *P63* | 72 | Male | T1 | 1 | Survival | 2012-02 | 1 | 7 | No |
| *P64* | 64 | Male | T1 | 1 | Death | 2008-05 | 1 | 6 | Yes |
| *P65* | 49 | Male | T1 | 1 | Survival | 2012-02 | 1 | 2 | No |
| *P66* | 50 | Male | T1 | 1 | Death | 2011-04 | 1 | 10 | Yes |
| *P67* | 54 | Male | T1 | 1 | Survival | 2012-02 | 1 | 1.2 | No |
| *P68* | 39 | Male | T2 | 2 | Death | 2008-12 | 1 | 10 | Yes |
| *P69* | 48 | Male | T2 | 2 | Death | 2010-09 | 1 | 3 | Yes |
| *P70* | 60 | Male | T1 | 1 | Survival | 2012-02 | 1 | 5.3 | No |
| *P71* | 56 | Female | T1 | 1 | Death | 2011-07 | 1 | 5 | Yes |
| *P72* | 45 | Female | T1 | 1 | Survival | 2012-02 | 1 | 2.2 | No |
| *P73* | 43 | Male | T1 | 1 | Death | 2008-10 | 2 | 2.5 | Yes |
| *P74* | 64 | Female | T1 | 1 | Death | 2009-08 | 1 | 6 | Yes |
| *P75* | 51 | Male | T1 | 1 | Death | 2010-08 | 1 | 2 | Yes |
| *P76* | 69 | Male | T1 | 1 | Death | 2010-10 | 1 | 5 | Yes |
| *P77* | 62 | Female | T1 | 1 | Death | 2010-10 | 2 | 5.5 | Yes |
| *P78* | 45 | Male | T1 | 1 | Survival | 2012-02 | 1 | 4 | No |
| *P79* | 64 | Male | T2 | 2 | Survival | 2012-02 | 1 | 3.6 | No |
| *P80* | 42 | Male | T1 | 1 | Survival | 2012-02 | 1 | 6 | No |
| *P81* | 49 | Male | T2 | 2 | Death | 2008-01 | 1 | 15 | Yes |
| *P82* | 41 | Male | T1 | 1 | Survival | 2012-02 | 1 | 2 | No |
| *P83* | 55 | Male | T2 | 2 | Death | 2008-03 | 2 | 14 | Yes |
